# DNA methylation profiling in Huntington’s disease reveals disease associated changes in the striatum

**DOI:** 10.1186/s13148-026-02082-4

**Published:** 2026-05-26

**Authors:** Gregory Wheildon, Adam R. Smith, Luke Weymouth, Joshua Harvey, Morteza Kouhsar, Lachlan F. MacBean, Claire Troakes, Ehsan Pishva, Rebecca G. Smith, Katie Lunnon

**Affiliations:** 1 Department of Clinical and Biomedical Sciences, Faculty of Health and Life Sciences, University of Exeter, Exeter, UK; 2 Institute of Psychiatry, Psychology & Neuroscience (IoPPN), King’s College London, De Crespigny Park, London, UK; 3 Department of Psychiatry and Neuropsychology, Mental Health and Neuroscience Research Institute (MHeNS), Maastricht University, Maastricht, The Netherlands

**Keywords:** Brain, Cerebellum, DNA methylation, Epigenetics, Epigenome-wide association study (EWAS), Entorhinal cortex, Huntington’s disease (HD), Illumina infinium methylation EPIC v1.0 array, Striatum, Weighted gene correlation network analysis (WGCNA)

## Abstract

**Background:**

Huntington’s disease is caused by a trinucleotide CAG repeat expansion in the *HTT* gene. Despite displaying autosomal dominance, phenotypic variation exists amongst mutation carriers, in particular relating to the age that symptoms first occur. This variation is primarily driven by an inverse relationship between CAG expansion size and age of symptom onset. However, the majority of variation in age of onset that is independent of CAG repeat length is thought to be driven by environmental influences. Since DNA methylation can be altered by environmental factors, and as methylomic variation is reported in other neurodegenerative diseases, it may offer a potential mechanism underlying disease manifestation.

**Results:**

We utilized the Illumina EPIC v1 methylation array to profile DNA methylation in 120 samples, including three distinct brain regions (striatum, entorhinal cortex and cerebellum) in 20 Huntington’s disease and 22 control donors. We identified seven Bonferroni-significant differentially methylated CpGs within the striatum along with 27 differentially methylated regions, annotated to genes involved in physiological processes known to be disrupted in HD such as the urea cycle and metabolism. Weighted gene correlation network analysis identified modules of co-methylated CpGs that were associated with Huntington’s disease, with ontological analyses showing enrichment in disease relevant processes. Furthermore, integration of single-nuclei RNA sequencing data highlighted that genes annotated to these modules are enriched in striatal spiny projection neurons, the primary cell types affected in the disease.

**Conclusions:**

Here, we present the first epigenome-wide association study of Huntington’s disease conducted in the striatum, the primary region of neuropathology, along with matched entorhinal cortex and cerebellum on the Illumina EPIC v1 array. Our results suggest that DNA methylation is altered at loci associated with Huntington’s disease in disease relevant regions and cell types and strengthens evidence for areas of potential therapeutic intervention.

**Supplementary Information:**

The online version contains supplementary material available at 10.1186/s13148-026-02082-4.

## Background

Huntington’s disease (HD) is a neurodegenerative condition caused by an autosomal dominant trinucleotide repeat expansion of a CAG motif in exon one of the *HTT* gene [1]. This results in a multifactorial phenotype, primarily defined by disordered movement, but also characterized by cognitive deficits and psychiatric disturbances [2].

The primary sites of pathology within the brain are the basal ganglia, in particular, severe neurodegeneration within the major structures of the striatum [3]. This occurs through the destruction of GABAergic striatal spiny projection neurons (SPNs) [4]. However, disease associated changes are not just restricted to the striatum. Cortical areas, including the entorhinal cortex, show reduced volume in early-mid stage disease that is linked to cognitive changes [5], and heavy neuronal loss has been documented in the region [6]. Cerebellar atrophy correlates with motor symptoms; however, cerebellar Purkinje neuron loss is only observed in individuals with a predominantly motor phenotype [7], suggesting cerebellar involvement may only occur within a select population of HD patients.

Although differences in cognition can be observed in pre-manifest and early HD [8], the development of motor symptoms is the accepted standard measure of disease manifestation. The primary source of variation in age of motor onset between individuals is the length of the CAG repeat expansion, which displays an inverse correlation with symptom development [9]. CAG expansions of more than 35 repeats are pathogenic, however there is lower penetrance in individuals with less than 40 repeats and they tend to develop motor symptoms later in life [10]. This is in stark contrast to individuals with longer repeat lengths, as 40 or more repeats is nearly fully penetrant by the age of 70 [10].

In a large Venezuelan kindred study, a mean repeat length of 45.72 resulted in symptom onset between 21 and 50 years of age, whilst individuals with a mean repeat length of 60.15 all developed symptoms before the age of 20 [11]. However, large differences between individuals are observed at any particular repeat length [12], with increased variation seen at lower pathogenic repeats [11, 12]. Therefore, other factors, both genetic and environmental, are suggested to contribute to disease manifestation [11]. Several genetic modifiers have been described from genome-wide association studies (GWAS), including single nucleotide polymorphisms (SNPs) in genes associated with DNA repair [13, 14], and disruption to the CAG repeat expansion in *HTT* itself [15]. Despite these genetic factors, the largest contribution to non-CAG repeat length related variation in age of onset comes from environmental factors [11].

Epigenetic processes are one mechanism by which the environment can regulate gene expression. The most well characterized epigenetic mechanism in neurodegenerative disease is DNA methylation [16–21]. The addition of a methyl group to the 5th carbon of cytosine (5mC) in a CpG dinucleotide is usually associated with gene silencing, although depending on the genomic context it has also been reported to increase expression or lead to alternative splicing [22]. An HD epigenome-wide association study (EWAS) of human post-mortem brain tissue, conducted using the Illumina Infinium 450K methylation array (450K), reported a substantial number of differentially methylated positions (DMPs) in a meta-analysis of the frontal, parietal and occipital cortices [23]. The authors noted p-value (*P*) inflation, as well as methodological issues related to intra-individual sampling, however, overall HD status was associated with an epigenetic age acceleration. Surprisingly, the severity of HD pathology was not associated with a summative increase in epigenetic age acceleration, with severe cases displaying a slowing of age acceleration and even deceleration in the most severe cases [23].

Another EWAS using the 450K array and restricted to a very small sample size (N = 7 HD cases), found no significant DMPs in the frontal cortex but observed a correlation between a substantial proportion of the overall variation in DNA methylation and age of onset [24]. The first EWAS in HD using the more recent Illumina Infinium EPIC methylation array (EPIC) was conducted in blood from over 1,600 individuals and found 33 CpG sites that showed significant differential methylation, including a site in the *HTT* gene [25]. Hypermethylation was observed at this site in the *HTT* gene in several brain regions when leveraging existing 450K data [25]. This change was not observed in the caudate nucleus, despite the prominent role striatal pathology has in HD, although this may be reflected by changes in cell proportions due to neuronal loss [3, 25].

**Fig. 1:**
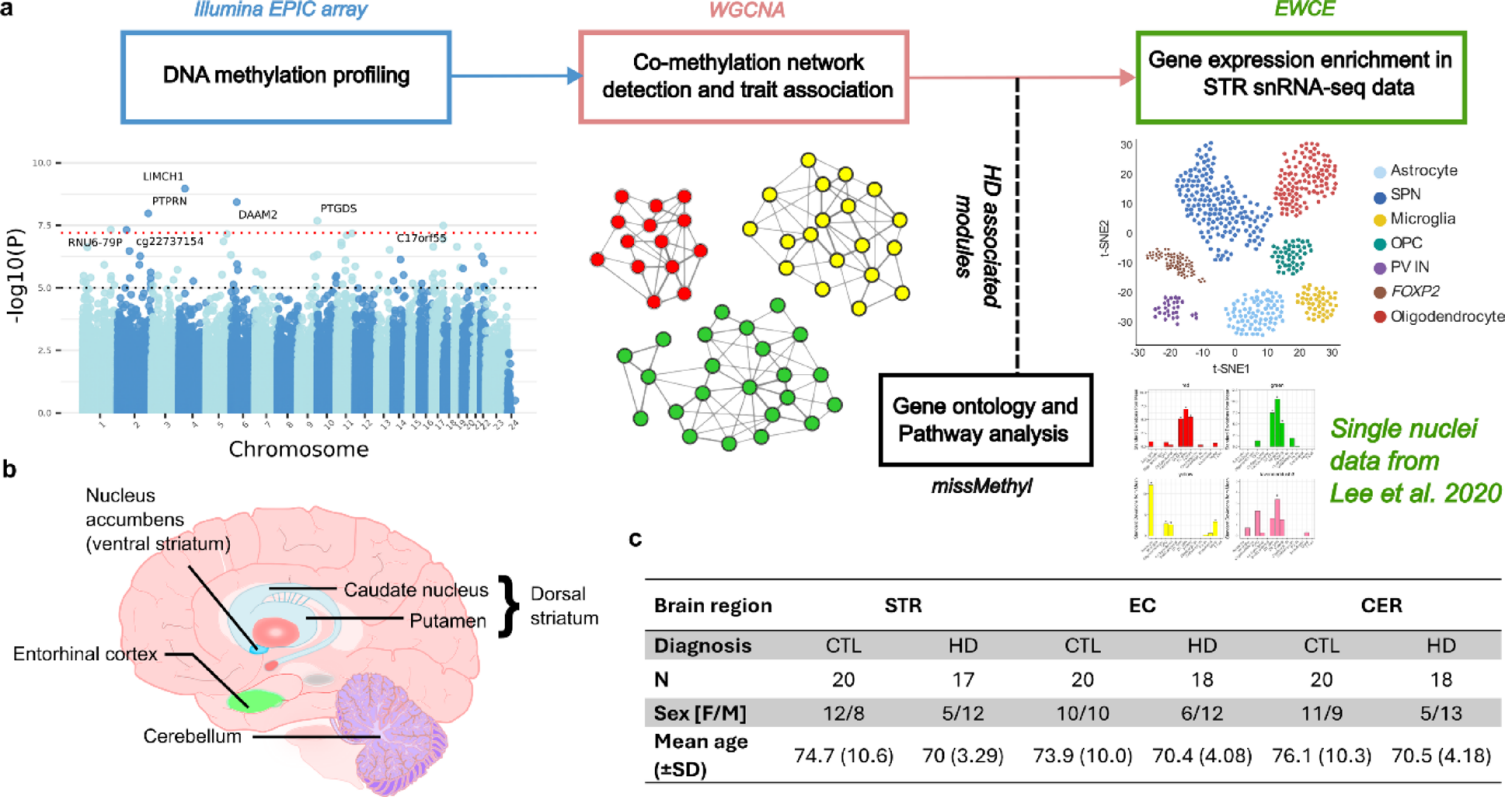
Overview of experimental design and sample cohort. **a** Data analysis flow chart as detailed in Methods. Single nuclei data used in EWCE analysis was taken from Lee et al. [45]. Representative UMAP plot created with Biorender. **b** Brain sagittal view displaying brain regions profiled in the study. **c** Summary table of the final sample cohort (after data quality control) by brain region: striatum (STR), entorhinal cortex (EC) and cerebellum (CER). Samples were split into two groups, control (CTL) and HD. Standard deviation (SD) is displayed in brackets next to the mean age

To date, all the EWAS conducted in post-mortem brain tissue taken from HD patients have used the 450K array [23, 24]. Indeed, the only HD methylation study conducted on the EPIC array using brain-like samples examined HD fibroblast-derived, induced neurons and showed these cells had an accelerated epigenetic age [26]. Therefore, we sought to profile DNA methylation in human HD brain tissue on the EPIC array, due to the increased genomic coverage the platform offers, in brain regions not previously subjected to EWAS that are affected by the disease: the striatum, entorhinal cortex and cerebellum. We utilized a two-pronged approach to explore DNA methylomic signatures in HD brain: an EWAS to identify DMPs associated with disease, and gene network correlation analysis to identify groups of co-methylated CpGs associated with disease, with subsequent ontological and single cell enrichment analyses (Fig. 1).

## Methods

### Subjects and samples

For our HD EWAS we selected a cohort of 42 individuals; 20 had a clinical and pathological diagnosis of HD and 22 were non-diseased controls with no significant neuropathology, utilizing three matched brain regions (striatum, entorhinal cortex, cerebellum). All three brain regions were profiled where available, giving a total sample size of 120. The samples were acquired from four UK brain banks (the Cambridge Brain Bank (CBB), the London Neurodegenerative Diseases Brain Bank (LNDBB), the Manchester Brain Bank (MBB) and the Oxford Brain Bank (OBB)) and all were dissected by trained professionals, snap-frozen and stored at − 80 °C. Further details on sample demographics are shown in Supplementary Table 1. DNA was extracted from 100 mg brain tissue using a standard phenol-choloroform extraction method and tested for degradation and purity, as previously described [27]. 500 ng of DNA from each sample was sodium bisulfite-treated to allow DNA methylation profiling using the Zymo EZ-96 DNA Methylation-GoldTM Kit (Cambridge Bioscience, Cambridge, UK) according to the manufacturer’s instructions.

### Illumina EPIC array profiling and data quality control

Samples were profiled using the Illumina Infinium Methylation EPIC v1.0 array (Illumina, San Diego, CA, USA), according to the manufacturer’s instructions, and DNA methylation was quantified using the Illumina iScan System (Illumina, San Diego, CA, USA). The samples were randomized with respect to tissue, sex, and disease status to avoid batch effects. Raw signal intensities generated for each probe were extracted using Illumina Genome Studio software.

All computational and statistical analysis were performed using R 4.2.1 [28] and Bioconductor 3.16 [29]. Signal intensities were imported into R as a methylumi object and RGChannel set object using the *methylum*i [30] and *minifi* [31] packages, respectively. Unless otherwise stated, quality control (QC) metrics were assessed using the *wateRmelon* package [32]. Samples were excluded from further analysis if: (1) either the median methylated or unmethylated fluorescent signal intensities was < 1000; (2) the bisulfite conversion rate was < 80%; (3) there was a discordance in the reported sex and the observed sex, as reported by the *minifi* package; (4) the maximum correlation, as calculated by pairwise complete observation between the 59 SNP probes on the array, was < 0.9 for matched samples; or (5) a genetic correlation of > 0.8 was seen between unmatched samples. Further sample and probe exclusion was performed using the *pfilter()* function in *wateRmelon* with the following thresholds: samples with a detection *P* > 0.05 in > 5% probes, probes with a beadcount < 3 in > 5% of samples, and probes with a detection *P* > 0.05 in > 1% samples. The final sample exclusion step was performed using the *outlyx()* function in *wateRmelon* to detect outlying samples. Cross-hybridizing probes, the 59 SNP probes, and probes that contained SNPs with a minor allele frequency (MAF) > 5% in the CG or single base extension position were excluded from downstream analysis [33]. This resulted in a total of 797,256 probes and 113 samples passing QC (Supplementary Table 1). The *dasen* function in *wateRmelon* was used to quantile normalize the data [32], with normalization performed separately for each brain region.

### Epigenome-wide association study

Principal component analysis (PCA) was then used to assess variation in the DNA methylation data using the *prcomp* base R function, with principal components (PCs) correlated with co-variates to identify confounders to control for in the subsequent analyses (Supplementary Fig. 1). Linear regression models were used to explore the association of DNA methylation with respect to HD status, controlling for the co-variates of sex, age, neuronal/glia proportion, bisulfite conversion plate and brain bank (modelled as separate co-variates). The CETS package was used to calculate the proportions of neuron/glia in the cortical samples [34], but was not used for the cerebellum samples as NeuN (which was used to generate the CETS algorithm) is not expressed by Purkinje neurons, the dominant cell type in the cerebellum [35]. Quantile–quantile (QQ) plots were used to assess the models for inflation, with the *bacon* R package used to remove observed inflation in the striatum and entorhinal cortex [36]. Subsequently the lambda values for all models were < 1.2 (Supplementary Fig. 2). The genome-wide significance threshold was defined as Bonferroni (*P* < 6.27 × 10^–8^) in line with previous EWAS [21, 37–39]. To identify differentially methylated regions (DMRs) consisting of ≥ 3 spatially correlated CpG sites, the Python module *comb-p*, run through the command line, was applied to the data, using a sliding window of 500 base pairs (bp) [40].

### Genomic enrichment analysis

We utilized Brown’s method of combining *P*-values to examine whether HD-associated methylation was enriched in genomic regions associated with HD motor symptom age of onset as identified in a GWAS by Lee et al. 2019 [15]. Forty-five genome-wide significant regions (*P* < 2.5 × 10^–6^) were tested and the region defined using a 35 kb upstream and 10 kb downstream window of each gene as per Lee et al. 2019. Of these, 44 contained > 1 CpG site on the EPIC array, and the *P*-values for the CpGs within a region were combined using the *Empirical Brown’s method* package [41], which accounts for intra-probe correlation.

### Weighted gene correlation network analysis (WGCNA)

The *WGCNA* R package was used to identify clusters of highly correlated CpG sites (modules) [42]. First, linear regression was used to remove the variance associated with the covariates used in the EWAS (i.e., age, sex, plate, brain bank in all brain regions, as well as neuron/glia proportions in striatum and entorhinal cortex samples) from the normalized data, by extracting the model residuals, which were then scaled by adding the intercept coefficient. Next, non-variable probes were removed (i.e., variance < median variance in a brain region), leaving 482,871 probes for module generation. Outlier samples in each dataset were assessed using Euclidean distance clustering and PC correlations, with five, four and five outliers removed from the striatum, entorhinal cortex and cerebellum datasets, respectively. To create the networks, *WGCNA* applies a weighting to the co-regulated similarity between loci through the selection of a soft threshold [42]. The scale free topology was plotted against the soft-thresholding powers, and the lowest power with a median connectivity of k < 25 was chosen: 11 for the striatum and 12 for the other two brain regions. To construct the network and generate the modules, the *blockwiseModules* function was used (unsigned network, min size = 100, max size = 10,000, deepSplit = 0).

### Association of modules with traits

Modules were arbitrarily assigned a color label, with the grey module containing all unassigned probes. The module eigengene (ME) is the first PC of the DNA methylation values of the probes within a module and represents the methylation profile of the module. The MEs for each brain region were correlated with variables to determine their association, using Spearman’s correlation for binary variables (e.g., disease status) and Pearson’s correlation for continuous variables (e.g., age). Modules were filtered to remove the grey module and any modules retaining any significant association with confounding variables. The remaining modules were used to calculate the Bonferroni correction level as follows: 0.05/number of modules. This resulted in correction levels of *P* < 1.09 × 10^–3^, *P* < 2.38 × 10^–3^ and *P* < 3.13 × 10^–3^ for the striatum, entorhinal cortex and cerebellum, respectively. For the modules showing a significant association with disease status, we calculated the module membership (MM) (Pearson’s correlation between a probe’s DNA methylation value and the ME value of its assigned module) and probe significance (PS) (Spearman’s correlation between a probe’s DNA methylation value and HD status).

### Gene ontological enrichment analysis

Gene Ontology (GO) and Kyoto Encyclopaedia of Genes and Genomes (KEGG) pathway analyses were conducted using the gene lists annotated to the probes within modules with a significant association with HD. For modules with more than 1000 probes, hub probes, defined as those probes with a MM > 0.8 and a PS < 0.05, were used in the pathway analysis. The background gene list was generated from all 482,871 probes used to generate the modules. Pathway analysis was performed by utilizing the GO and KEGG repositories through the *gometh* function in the *missMethyl* package [43], and this method was selected as *gometh* adjusts for the number of CpG sites within a gene. As similar ontology terms are observed in GO analysis due to overlapping gene sets, modules were merged based on semantic similarity using the *rrvgo* package [44]. The Resnik’s measure was used to compute term similarity, with a medium between terms similarity of 0.7 selected. Due to a large number of returned GO terms, we restricted reported terms to those with an uncorrected *P* < 0.01, whilst for KEGG terms we reported all terms reaching nominal significance (*P* < 0.05).

### HD genetic modifier enrichment analysis

We utilized the same genomic regions identified by Lee et al. [15] as in our genomic enrichment analysis, to test if probes within these regions were enriched in significant HD-associated modules, identified by WGCNA. Fisher’s exact test was used to test for enrichment of CpGs using a background size of N = 482,871, corresponding to the number of CpGs used for WGCNA module generation.

### Cell enrichment analysis

The annotated gene lists generated from the significant HD-associated WGCNA modules in the striatum were assessed for cell type enrichment using human single nuclei RNA sequencing (snRNA-seq) data generated in the striatum by Lee et al. 2020 with the 10X genomics platform (v3 Kit) [45]. Filtered single nuclei barcodes (with corresponding cell annotation UMAP and metadata), expression matrix, and gene feature files, were downloaded from the Gene Expression Omnibus (GEO) (GSE152058). The *Seurat* R package (version 5.0.3) was used to load the data via the *Read10X()* function. The *SummarizedExperiment* R package (version 1.28.0) was used to create a summarized experiment (SE) object from the data through the *SummarizedExperiment()* function. The UMAP cell type annotations, generated by the authors, were designated to the nuclei using colData when creating the SE object, and 33,538 profiled genes were used in downstream processing. This was performed using the Expression Weighted Cell Type Enrichment (*EWCE)* package [46] (version 1.6.0). Non-expressed genes (N = 3,036) and genes that were not significantly differentially expressed between cell types (N = 2,521, Benjamini-Hochberg (BH) adjusted *Q*-value threshold (*Q*) < 1 × 10^–5^) were removed with the *drop_uninformative_genes()* function, using the Limma setting with the input species set to ‘human’. The *generate_celltype_data()* function was used to calculate a normalized mean expression and specificity cell type dataset. The dataset was then examined visually using the *plot_ctd()* function to ensure that known marker genes displayed appropriate expression profiles in expected cell types (Supplementary Fig. 3). Methylated loci from HD-associated WGCNA modules in the striatum were used to generate individual annotated gene lists which were then tested for cell type enrichment using the *bootstrap_enrichment_test()* function, set to 100,000 repetitions. A BH corrected *Q* < 0.05 indicated significant enrichment in a cell type within that module. To check concordance in cell type enrichment between species, the same protocol was applied to a R6/2 HD mouse striatum snRNA-seq dataset, generated by the same researchers [45], available to download from GEO (GSE152058). All steps were the same as for the human dataset, except for parameters relating to species input, which were altered to ‘mouse’. The generated SE object contained 31,053 genes, 4,754 non-expressed genes and 4,175 non-significant differentially expressed genes were then removed before cell type dataset generation. The marker gene expression in the mouse cell type dataset was examined visually to ensure appropriate expression profiles in cell types (Supplementary Fig. 4).

## Results

### DNA methylation signatures in HD brain samples

**Fig. 2:**
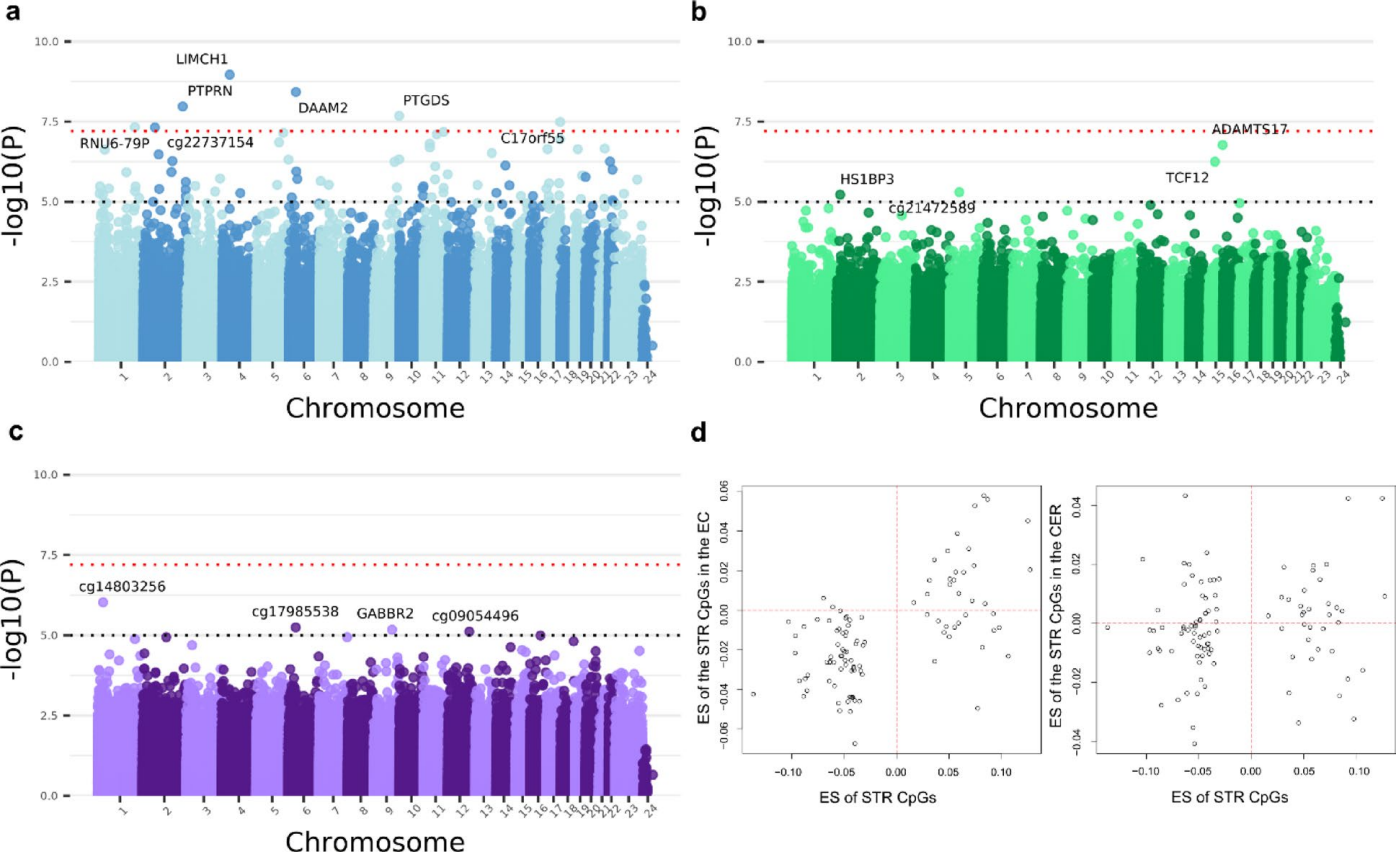
Alterations in DNA methylation are associated with HD status in the striatum. Manhattan plots of association between HD status and DNA methylation in the **a** striatum, **b** entorhinal cortex and **c** cerebellum. Bonferroni significant sites in the striatum and the most significant sites in the entorhinal cortex and cerebellum are annotated with the Illumina UCSC gene name, or the CpG ID if unannotated. The x-axis shows the chromosome number, with the X and Y chromosomes represented by 23 and 24, respectively. The y-axis shows –log10(*P*) and the dotted red line denotes the Bonferroni significance level (*P* < 6.27 × 10^–8^). **d** Scatter plots of the effect size (ES) of the 100 most significant CpG probes in the striatum (STR) (x-axis) correlated with the ES of the same probes in the entorhinal cortex (EC) (left panel), and the cerebellum (CER) (right panel) (y-axis). Probes in the bottom left and upper right quadrants, as denoted by the dotted redlines, indicate CpGs with the same direction of effect between the brain regions

Our EWAS identified seven genome-wide significant DMPs associated with HD in the striatum (*P* < 6.27 × 10^–8^) (Fig. 2a) (Supplementary Table 2). Of the seven Bonferroni-significant sites, all displayed hypomethylation in HD compared to control, with the exception of cg22300346, annotated to *PTPRN* (Supplementary Fig. 5). Three of the CpG sites were annotated to within 1500 bp of the transcriptional start site (TSS) of the associated gene (CpGs annotated to *LIMCH1*, *PTPRN* and *C17orf55*), whilst the site annotated to *PTGDS* was within 2000 bp of the TSS and the CpG annotated to *DAAM2* was located in the 5’ untranslated region (5’ UTR) (Supplementary Table 2). This suggests that five of the seven Bonferroni significant sites in the striatum were located in regulatory regions of the annotated genes. None of these seven loci were Bonferroni significant in the entorhinal cortex or cerebellum.

We observed no Bonferroni significant DMPs in the entorhinal cortex (Fig. 2b, Supplementary Table 3), or in the cerebellum(Fig. 2c, Supplementary Table 4). To assess whether there may be global methylation alterations in HD we also calculated the corrected global methylation mean of all CpGs, in each brain region, for the control and HD groups, and found no significant difference between the groups (t-test, striatum *P* = 0.951, entorhinal cortex *P* = 0.212 and cerebellum *P* = 0.990) (Supplementary Fig. 6).

### Effect sizes of the most significant striatal loci correlate with those in the entorhinal cortex and are observed in an independent dataset

Given the lack of Bonferroni significant HD-associated CpG sites in both the entorhinal cortex and the cerebellum, we sought to examine whether HD-associated changes observed in the striatum were also seen in the other brain regions. The effect sizes (ES) of the 100 most associated DMPs identified in the striatum were significantly correlated with the ES of those same sites in the entorhinal cortex (Pearson’s correlation, *r* = 0.62, *P* = 5.87 × 10^–12^) (Fig. 2d), with an enrichment for the same direction of effect (sign test: *P* = 1.31 × 10^–11^). Of the seven Bonferroni significant loci we identified in the striatum, all except cg22300346 (annotated to *PTPRN*) showed the same direction of effect in the entorhinal cortex. However, there was no correlation between the ES of these striatum DMPs with the ES in the cerebellum (Pearson’s correlation, *r* = 0.136, *P* = 0.179) (Fig. 2d), and although there was a weak enrichment for the same direction of effect (sign test: *P* = 0.021), the lack of correlation suggests that any methylation changes occurring in this region were distinct from those we identified in the other brain regions.

We were interested in exploring whether our observations in the striatum showed concordance with existing HD DNA methylation datasets. Therefore, we investigated the overlap between our 100 most associated striatum CpGs and the CpG sites identified by Horvath and colleagues across multiple brain regions in 26 HD and 21 control donors that they had profiled using the 450K array [23]. Of the 100 most associated striatum CpGs we had identified, 48 were present in the summary statistics from the meta-analysis of frontal, occipital and parietal brain regions performed by Horvath and colleagues. For these 48 sites, we observed a significant correlation of our striatum ES with the Z-scores (denoting methylation effect) reported in the Horvath et al. meta-analysis (Pearson’s correlation,* r* = 0.611, *P* = 3.94 × 10^–6^), and an enrichment for the same direction of effect (sign test: *P* = 3.31 × 10^–6^) (Supplementary Fig. 7). Taken together, this suggests that the methylation changes we observed in the striatum are also present in cortical brain regions and are replicated in independent HD brain DNA methylation datasets.

### Multiple differentially methylated regions are associated with HD in the striatum

**Fig. 3:**
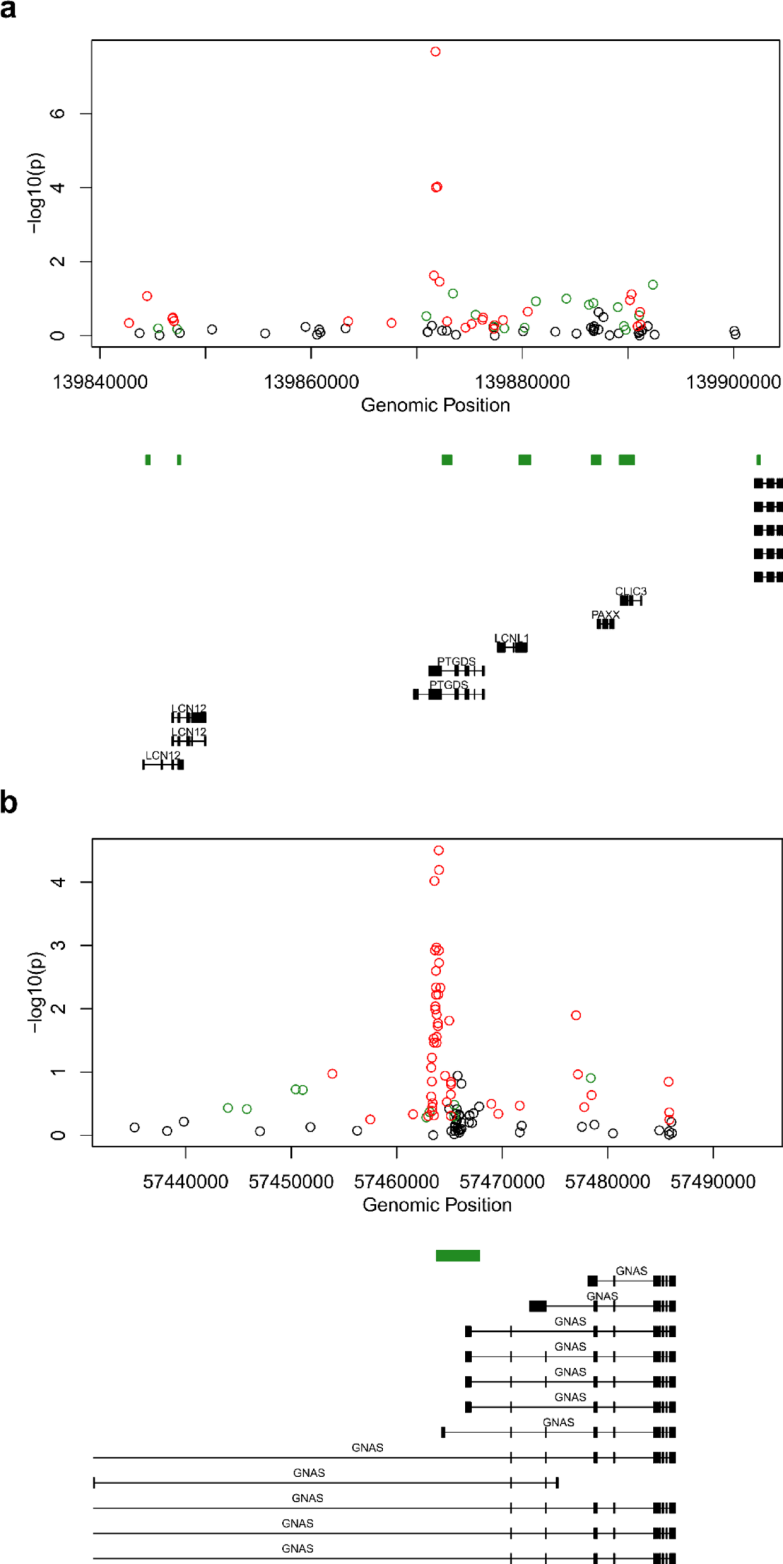
The top DMRs associated with HD in the striatum and cerebellum display hypomethylation. Mini-Manhattan plots for the most significant DMRs containing at least three probes in **a** the striatum (*PTGDS*) and **b** the cerebellum (*GNAS*). Green probes (circles) represent a positive effect size (ES) ≥ 0.01, red probes (circles) represent a negative ES ≤ -0.01. 500 bp windows were used to designate DMRs. The x-axis shows the genomic position. The y-axis shows the –log10(*P*). Underneath, the gene tracks are shown in black with CpG islands in green

To identify DMRs consisting of multiple neighbouring DMPs, *comb-p* analysis was conducted [40]*,* which revealed 27 significant striatum DMRs spanning at least three probes (Supplementary Table 5). The most significant DMR was a hypomethylated region, consisting of four probes annotated to *PTGDS* (Šídák -corrected *P* = 2.21 × 10^–8^), containing the fourth most significant DMP from the EWAS (Fig. 3a). Two other highly significant regions included a ten probe DMR in *RHCG* (Šídák -corrected *P* = 5.95 × 10^–8^) and a nine probe DMR in *COL18A1* (Šídák -corrected *P* = 9.39 × 10^–8^). These regions also displayed hypomethylation, and, to our knowledge, none of the genes housing the top three DMRs had previously been associated with HD. Given that the *PTGDS* DMR also contained one of the Bonferroni-significant DMPs, we decided to correlate the methylation across the 17 CpG sites annotated to this gene to explore whether these were independent methylation signals. Of the 17 sites in the region on the EPIC array, nine showed a strong correlation with each other, including the probes in the DMR, indicating these sites may be part of a co-methylated block within the gene (Supplementary Fig. 8).

No significant DMRs were found in the entorhinal cortex. In the cerebellum, two DMRs were identified (Supplementary Table 6), including a 19 probe DMR in *GNAS* (Šídák -corrected *P* = 2.7 × 10^–16^) (Fig. 3b) and an eight probe DMR in *MEST* (Šídák -corrected *P* = 4.64 × 10^–5^). To our knowledge, neither region has previously been reported to be differentially methylated in HD. A shared characteristic of the annotated genes is that both are imprinted genes [47, 48], meaning only one parental allele is expressed. All of the probes within the DMRs in the cerebellum were hypomethylated in HD.

### DNA methylation variation in HD may be enriched in the vicinity of the *HTT* gene

We assessed whether HD-associated DNA methylation signatures were enriched in 45 genomic regions that had previously been associated with age of motor onset in HD by Lee et al. 2019, examining a window spanning 35 kb upstream and 10 kb downstream of the region defined by the Entrez ID [15]. Of these regions, 44 housed > 1 CpG site on the EPIC array and we used Brown’s method to combine the *P*-values of the sites within each of these regions. We observed very little enrichment in any of the three brain regions, with no regions passing Bonferroni correction (*P* < 0.00114) (Supplementary Table 7). However, we noted that the lowest combined *P*-value in the striatum was associated with the *HTT* region, which narrowly missed nominal significance (chr4:3041408–3255687, *P* = 0.0575). Whilst the *HTT* region itself did not show nominal enrichment in either the entorhinal cortex or cerebellum, in the entorhinal cortex nominally significant enrichment was observed at *GRK4* (chr4:2930232–3052474, *P* = 0.035). This region is upstream of *HTT* on chromosome 4 and there is approximately 10 kb overlap between the end of the *GRK4* region and the start of the *HTT* region tested, indicating possible genomic enrichment of methylation variation occurs in an extended region around *HTT* in multiple brain regions. In the cerebellum nominally significant enrichment was observed at *C3orf35* (chr3:37391477–37486988, *P* = 0.0286).

### DNA co-methylation networks are associated with HD in the striatum

**Fig. 4:**
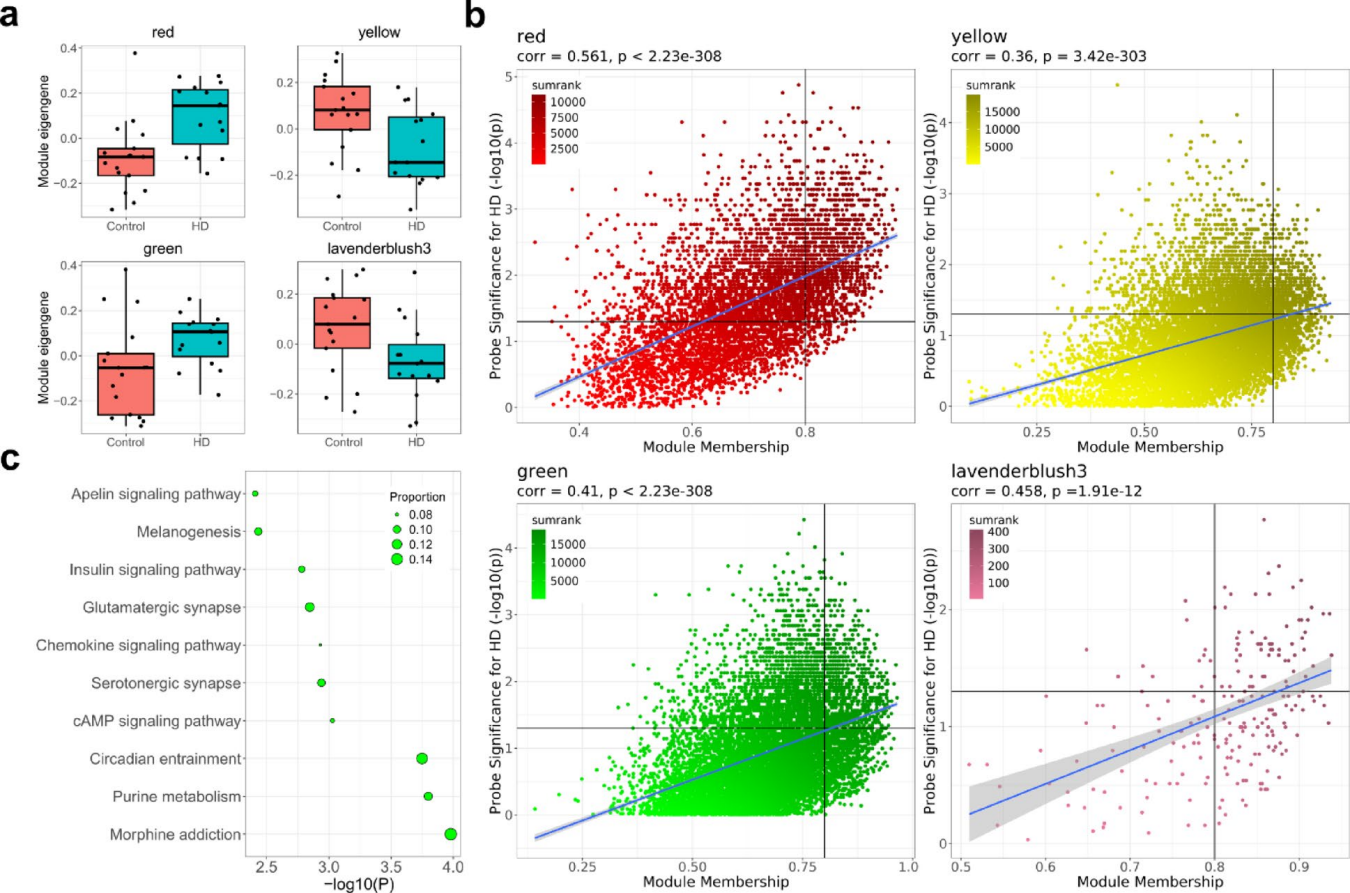
Highly connected methylation networks are strongly associated with HD in the striatum and are enriched in neuronal related pathways. **a** Boxplots of the ME value (y-axis) for the control and HD groups of the red module (*P* = 2.08 × 10^–3^), yellow module (*P* = 0.0168), green module (*P* = 0.0355) and lavenderblush3 module (*P* = 0.0389). The thick black line represents the median value. The boxes represent the middle 50% of values and the whiskers represent the 1st and 4th quartiles. **b** The module membership (MM) (x-axis) plotted against the probe significance (PS) (y-axis) for the same four modules. ‘sumrank’ refers to the summation of the rank each probe has for MM and PS, with a higher value indicating a stronger ranking. Each dot represents a CpG within the module. The blue lines represent the line of best fit determined by linear regression, with the grey shaded area representing the 95% confidence interval. **c** The ten most significant terms for KEGG pathway analysis of the hub probes (N = 731) of the green module, which was significantly associated with HD. The terms are arranged from least significant to most significant. The x-axis displays the -log10(*P*). Points are sized by the proportion of CpGs in the total sites annotated to that term that are part of the green module hub probes

WGCNA was used to identify modules of co-methylated probes in each of the three brain regions. After regressing out the co-variates, the modules were generated and subsequently tested for association with HD status and potential confounding variables. Co-methylated probes were clustered into 46 modules in the striatum, 21 modules in the entorhinal cortex, and 15 modules in the cerebellum, after module filtering to exclude non-variable probes and modules associated with confounders. Six modules in the striatum were significantly associated with HD (*P* < 0.05), which were the red (N = 5,577 probes), yellow (N = 9,972 probes), navajowhite2 (N = 296 probes), green (N = 9,617 probes), lavenderblush3 (N = 213 probes) and grey60 (N = 804 probes) modules. Although none of these passed the Bonferroni significance threshold the red module showed the highest significance (*P* = 0.004) (Supplementary Fig. 9). All six modules had a nominally significant difference between the ME values of the control and HD groups (t-test: *P* < 0.05), with the red module again displaying the strongest association, surpassing a BH adjusted threshold of *Q* < 0.1 (Fig. 4a; Supplementary Fig. 10). In both the entorhinal cortex and the cerebellum no modules showed a significant correlation with HD (Supplementary Figs. 11–12).

### Highly connected probes show strong association with HD status in the striatum modules

To further explore the relationship between the significant striatum modules and HD, the module membership (MM), which is a measure of the connectivity of a probe within a module, was correlated against PS, the significance an individual probe had in relation to the trait of interest (i.e., HD). Whilst all the modules exhibited a significant correlation, when correlating the absolute MM against the -log_10_ probe significance, only the red (Pearson’s Coefficient (*r*) = 0.561, *P* < 2.23 × 10^–308^), yellow (*r* = 0.36, *P* < 3.42 × 10^–303^), green (*r* = 0.41, *P* < 2.23 × 10^–308^) and lavenderblush3 (*r* = 0.458, *P* = 1.91 × 10^–12^) modules showed a moderate correlation (*r* > 0.3) (Fig. 4b). We therefore focused on these modules for subsequent downstream analyses. When plotting this relationship, there were a large proportion of probes with a high summated ranking of the two measures clustered in the upper right of each plot, indicating both a high MM and high PS (Fig. 4b, Supplementary Tables 8–11). Indeed, the number of hub probes for each module, defined as a probe with a MM of > 0.8 and a PS *P* of < 0.05, was 1,064 for red (19.1% of total probes), 454 for yellow (4.55% of total probes), 731 for green (7.6% of total probes) and 59 for lavenderblush3 (27.7% of total probes). We sought to further characterize the hub probes of each module by examining whether multiple hub probes were annotated to the same genomic region, termed hub genes. Each module contained one or more hub genes with at least two probes annotated to it (Supplementary Table 12).

### Annotated genes in HD associated modules show ontological enrichment for disease-relevant processes.

To explore the potential biological relevance of the HD associated striatum modules we performed GO and KEGG pathway analysis on the genes annotated to the probes within the modules [43]. For modules containing over 1,000 probes (i.e., red, yellow and green modules) the analysis was restricted to the hub probes in the module, whereas for smaller modules (i.e., lavenderblush3 module) the analysis was conducted on the entire module. The red module hub probes (N = 1,064 CpGs) showed enrichment for 32 GO terms at *P* < 0.01, although none passed false discovery rate (FDR) correction (Supplementary Fig. 13a; Supplementary Table 13). The most significant term related to hematopoietic or lymphoid organ development; however, two of the ten most significant terms related to purinergic signalling and one term related to hindbrain development. Nominal enrichment was found for 13 KEGG pathways in the red module hub probes and several of these related to important cell signalling pathways (Rap1, Tumor necrosis factor (TNF), mTOR, Gonadotropin-releasing hormone (GnRH)). Several neuronal terms were also reported including cholinergic synapse, long-term depression and glutamatergic synapse (Supplementary Fig. 13b; Supplementary Table 14).

Within the yellow module hub probes we identified 35 GO terms at *P* < 0.01 (Supplementary Fig. 14a; Supplementary Table 15), with the most significant term relating to cellular response to alcohol, whilst several of the most significant terms related to neuronal development. Of the 70 nominally significant KEGG pathways that we identified in the yellow module hub probes, many of the most significant were related to neuronal function, including long-term potentiation, glutamatergic synapse, and circadian entrainment. (Supplementary Fig. 14b; Supplementary Table 16).

We identified 56 GO terms at *P* < 0.01 in our analysis of the hub probes in the green module (Supplementary Fig. 15; Supplementary Table 17), with the most significant terms all displaying potential biological relevance to HD, for example nucleotide/nucleoside metabolism, scaffold protein binding, hippocampal signalling, somatodendritic compartment, AMPA glutamate receptors, and transmembrane transport. For the KEGG pathway analysis 34 terms were nominally significant, including three terms at FDR significance (*Q* < 0.05): morphine addiction, purine metabolism and, the neuronal related term, circadian entrainment (Fig. 4c; Supplementary Table 18).

As the lavenderblush3 module contained 213 probes the entire module was used for pathway analyses, with 51 GO terms at *P* < 0.01 (Supplementary Fig. 16a; Supplementary Table 19). Two of the top ten related to the renal system, one to synaptic vesical coating, and several were related to G protein-coupled signalling. Fifteen KEGG terms were nominally enriched in the module, and whilst the top ten most significant displayed disparate terms related to processes such as lipolysis regulation, growth hormone action, vitamin absorption and cocaine addiction, expansion to all 15 terms returns two more related to addiction (alcoholism and morphine addiction) as well as the neuronal related term, long-term depression (Supplementary Fig. 16b; Supplementary Table 20).

### HD co-methylated networks are mostly independent of HD associated genetic variation

To further examine the biological relevance of the HD associated modules, we used two-sided Fisher’s exact tests to test the enrichment of the CpGs within each module in genetic regions previously identified in GWAS as genetic modifiers of HD age of onset [15]. Of the genetic modifier regions, probes annotated to *LETM1* in the red module (odds ratio (OR) = 3.36, *P* = 0.035) (Supplementary Table 21), *FAM193A* in the yellow module (OR = 4.31, *P* = 0.0172) (Supplementary Table 22) and *ANKRD34B* in the green module (OR = 6.15, *P* = 0.0491) (Supplementary Table 23) showed a nominal enrichment, whilst no enrichment was observed for any of the GWAS regions in the lavenderblush3 module. Taken together these results indicate that the co-methylated networks we have identified as being associated with HD, are largely independent of genetic variation associated with HD age of onset.

### Genes annotated to HD-associated modules have significantly enriched expression in disease affected neuronal subtypes in the striatum

**Fig. 5:**
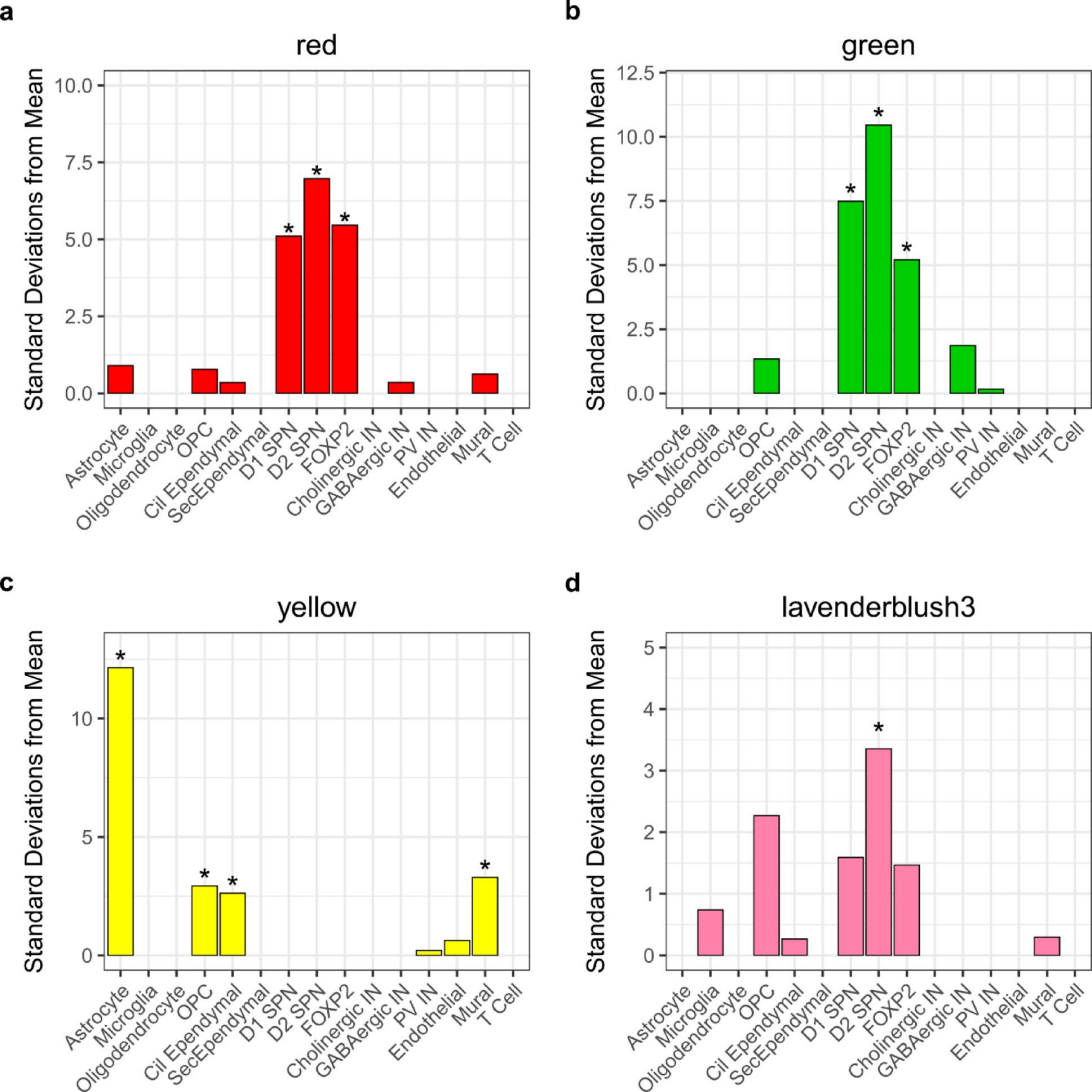
Genes annotated to the green and red module hub probes are enriched in striatal neurons affected in HD. Weighted cell type enrichment analysis of the genes annotated to the hub probes in the **a** red, **b** green and **c** yellow modules, and all probes in the **d** lavenderblush3 modules, leveraging a human striatum snRNA-seq dataset obtained from Lee et al. [45]. The y-axis displays the cell type: IN = interneuron, PV = Parvalbumin, Cil Ependymal = cilia ependymal cells and Sec Ependymal = secretory ependymal cells. FDR enrichment (*Q* < 0.05) is denoted with an asterisk. The x-axis displays the number of standard deviations that the mean expression for the genes in each module is, relative to the bootstrapped mean for the particular cell type

Given that our pathway analyses on the HD-associated modules revealed a number of neuronal related terms, we were interested in exploring whether the co-methylated loci within the modules were annotated to genes known to be expressed in cell types affected by HD. To do this we used EWCE to test for cell type enrichment of the genes annotated to the red hub probes, the yellow hub probes, the green hub probes and the lavenderblush3 module probes, separately, leveraging a publicly available human snRNA-seq dataset generated in the striatum [45]. For the red module hub probes, 516 annotated genes overlapped with the striatum snRNA-seq dataset, and these showed an FDR significant enrichment in D2 dopamine receptor expressing (D2) SPNs (*Q* < 2.2 × 10^–16^)*,* D1 dopamine receptor expressing (D1) SPNs (*Q* < 2.2 × 10^–16^) and *FOXP2/OLFM3*-expressing striatal (*FOXP2*) neurons (*Q* = 1.0 × 10^–4^) (Fig. 5a; Supplementary Table 24a). For the green module hub probes, 418 annotated genes overlapped with the snRNA-seq dataset, with the same three cell types showing an FDR significant enrichment (D2 SPN: *Q* < 2.2 × 10^–16^, D1 SPN: *Q* < 2.2 × 10^–16^, *FOXP2*: *Q* = 5.0 × 10^–5^) (Fig. 5b; Supplementary Table 25a). For the yellow module hub probes, 259 annotated genes overlapped with the snRNA-seq dataset, and we observed an FDR significant enrichment for astrocytes (*Q* < 2.2 × 10^–16^), mural cells (*Q* = 0.0111), oligodendrocyte progenitor cells (*Q* = 0.0234) and cilia ependymal cells (*Q* = 0.0276) (Fig. 5c; Supplementary Table 26a). Finally, for the 137 genes that overlapped between the lavenderblush3 module and the striatal snRNA-seq dataset, an FDR significant enrichment was observed for D2 SPNs (*Q* = 0.018) (Fig. 5d; Supplementary Table 27a).

Given that SPNs are the primary affected cell type in HD [4] and as *FOXP2* SPNs are a recently identified, distinct subtype [45, 49], the cellular enrichment of the gene networks present in the red and green module hub probes provides evidence of strong functional relevance to HD. Astrocyte proliferation is also a key hallmark of HD pathology [50], therefore the strong astrocyte enrichment observed in the yellow module provides further support for this evidence. To ensure that the cell type enrichments we identified were highly specific to these four HD-associated modules, we also tested the cellular enrichment across the other 42 filtered modules we had initially identified in the striatum but had not been associated with phenotype. Reassuringly, only one, two, eight and three other modules showed an FDR significant enrichment for D1 SPNs, D2 SPNs, *FOXP2* SPNs, and astrocytes, respectively (Supplementary Fig. 17).

To examine whether the cell type enrichment was preserved across species, we applied EWCE to our annotated gene networks using a R6/2 HD mouse snRNA-seq dataset generated in the same study as the human dataset [45]. We observed very close concordance in cell type enrichments between the human and the mouse datasets for the red, green, yellow and lavenderblush3 modules. Of the 505 overlapping genes in the red module hub probes and the mouse snRNAseq dataset, three FDR significantly enriched cell-types were found: D2 indirect pathway SPNs (iSPNs) (Q < 2.2 × 10^–16^)*,* D1 direct pathway SPNs (dSPNs) (*Q* < 2.2 × 10^–16^), and *Foxp2* neurons (*Q* = 5.0 × 10^–5^) (Supplementary Fig. 18a; Supplementary Table 24b). iSPNs and dSPNs are broadly orthogonal to D2 SPNs and D1 SPNs, respectively, indicating the cellular enrichment of the annotated gene network in the red hub probes is preserved in the mouse model. For the 394 genes annotated to the green module hub probes that overlapped with the mouse snRNA-seq dataset, the same three cell types were also FDR significantly enriched (*Q* < 2.2 × 10^–16^) (Supplementary Fig. 18b; Supplementary Table 25b). Again, these findings indicate the cellular enrichment of the annotated gene network in the green hub probes is preserved in the mouse model. 245 genes overlapped between the yellow module hub probes and the dataset, and similar to the human analysis, astrocytes were found to be FDR significantly enriched (*Q* < 2.2 × 10^–16^) (Supplementary Fig. 18c; Supplementary Table 26b). Similarly to the human snRNA-seq dataset, we observed an FDR significant enrichment for iSPNs for the 100 genes that overlapped between the lavenderblush3 module and the mouse snRNA-seq dataset (*Q* = 0.0392) (Supplementary Fig. 18d; Supplementary Table 27b).

## Discussion

To the best of our knowledge, this study represents the first EWAS of HD brain tissue using the EPIC array, and the first EWAS of HD in the striatum, entorhinal cortex and cerebellum. The two previous studies interrogating genome-wide DNA methylation levels in the brain of HD patients were conducted using the 450K array and profiled frontal, parietal and occipital cortex tissue [23, 24]. We observed robust HD associated DNA methylomic variation in the striatum, with seven Bonferroni-significant CpGs, whilst less variation was observed in the entorhinal cortex and cerebellum. Despite this, similar DNA methylomic variation was still detected in the cortex, given the highly significant correlation between the 100 most associated CpGs in the striatum and those same CpGs in the entorhinal cortex in the same samples, suggesting that a larger sample size could have provided more power to identify Bonferroni-significant associations within the entorhinal cortex. Given that in our current sample size Bonferroni-significant associations were observed in the striatum, but not the cortex, this likely reflects that the striatum is the primary site of pathology in HD, being affected much earlier than cortical regions [3, 5]. Importantly, when we independently validated these methylation changes in the meta-analysis of frontal, parietal and occipital cortex previously performed on the 450K array by Horvath and colleagues [23], we observed a highly significant correlation of the effect size of the 48 overlapping sites. Together, this suggests robust and reproducible alterations in HD brain tissue across subcortical and cortical regions.

In an earlier study, Villar-Menéndez et al. reported DNA methylation changes in the adenosine A_2A_ receptor (*ADORA2A*) gene in human striatal tissue in HD [51]. However, De Souza and colleagues, in a latter study showed that when neuronal cell proportions were controlled for (via CETS) that there was no HD-associated differential DNA methylation in this gene in HD frontal cortex. The authors therefore suggested caution in interpreting DNA methylation changes in HD striatal tissue due to alterations in cellular proportions in the region [24]. Although our study utilized striatal tissue, we are confident that the loci we report are robust as we also used the CETS package to control for neuronal proportions in our striatum (and entorhinal cortex) tissue samples. Whilst our lack of genome-wide significant findings in cortical tissue is in apparent agreement with the De Souza study (but in disagreement with Horvath et al. [23]), it should be noted the De Souza et al. study was conducted in a very low sample size and used samples from across the frontal cortex, rather than a confined area (such as the entorhinal cortex in our case). This suggests that their study may have been limited by both lower power and greater sample heterogeneity, potentially impacting the detection of smaller effect sizes, which the authors themselves concede [24]. Moreover, in our study we observed significant correlations for the effect sizes of the top striatal CpG sites we had identified, with the effect sizes for the same loci in our matched entorhinal cortex samples, and importantly with the Z-scores in the Horvath study [23]. This highlights that the present work provides evidence to support the hypothesis that DNA methylation alterations occur within cortical brain tissue in HD, and that studies in larger and better-defined cohorts may strengthen this evidence further.

Of the genes annotated to the seven Bonferroni-significant CpGs we identified in the striatum, several had been previously associated with HD. The most significant DMP was annotated to *LIMCH1*, a regulator of the motor protein myosin II [52], and which is involved in the urea cycle [53]. Urea cycle disruption is known to be a feature in HD in brain regions including the striatum, entorhinal cortex and cerebellum. [54]. Importantly, *LIMCH1* has been shown to have increased gene expression in the striatum of a HD mouse model with a disrupted urea cycle phenotype, although the authors reported no change in protein expression [55]. The second most significant DMP was annotated to *DAAM2*, a gene involved in the developmental patterning of the spinal cord through regulation of the Wnt signalling pathway [56], which has been reported to have decreased expression in Human HD muscle tissue [57]. *PTPRN* encodes a transmembrane protein in dense core vesicles that regulates insulin secretion but is also thought to be involved in the regulation of neurotransmitter secretion [58, 59]. It has increased expression in striatal-like cells generated from HD patient induced pluripotent stem cell (iPSC) lines and the protein shows increased expression in the hippocampus of the R6/2 HD mouse model [60, 61]. *PTGDS* synthesizes prostaglandin D_2_ but also has a role in lipid transport [62]. *PTGDS* expression has been found to be decreased in oligodendrocyte and oligodendrocyte progenitor cell nuclei isolated from post-mortem brain tissue taken from HD patients, as compared to control samples [63]. This was observed in the caudate nucleus and cingulate cortex for both types of nuclei, and additionally in the nucleus accumbens for oligodendrocyte nuclei, with expression negatively correlated with CAG expansion length [63]. *PTGDS* has also be shown to bind amyloid-beta (Aβ), the key pathogenic peptide in Alzheimer’s disease (AD), by which it prevents Aβ aggregation in vitro, highlighting possible links between the gene and other neurodegenerative diseases[64]. *PTGDS* was also the most significant region we identified in our DMR analysis, spanning four probes in the transcriptional start site of the gene and the DMR contained the Bonferroni significant DMP we identified in the EWAS. When we explored methylation patterns in this gene further we identified nine probes that were strongly correlated with each other, including the four probe DMR, suggesting that these were co-methylated. In addition to *PTGDS*, we also identified 26 other DMRs in the striatum that passed Šídák correction. The second most significant DMR in HD striatum was annotated to *RHCG*, which encodes an ammonia transporter, further implicating the urea cycle. This gene has been previously reported to be upregulated in the striatum in an HD sheep model and was positively correlated with the expression of *SLC14A1,* the major urea channel [65], which the authors showed was upregulated in human post-mortem striatal HD tissue [65]. Together with the Bonferroni-significant DMP annotated to *LIMCH1*, this highlights potential alterations in urea metabolism in HD, and provides further support for the potential of therapeutically targeting urea cycle disruption in HD.

We sought to examine if DNA methylation was enriched in genomic regions which are associated with a phenotypic marker of HD, namely age of motor symptom onset [15]. Little genomic enrichment was observed in each of the profiled brain regions, however, in the striatum the *HTT* region narrowly missed reaching nominal significance, and in the entorhinal cortex a region annotated to *GRK4* showed nominal enrichment. This region is upstream of, and overlaps, the *HTT* region. A SNP on chromosome 4 in the *HTT* 5’UTR has been previously found to be associated with somatic expansion in HD blood [66]. The SNP is a cis-expression quantitative trait loci (eQTL) causing increased expression in of *GRK4* in whole blood [66, 67], therefore methylation variation in this region could also be under genetic influence. *GRK4* has increased expression in the striatum of a HD mouse model, where CAG expansion has been found to alter chromatin conformation in the region [68]. Altered chromatin conformation was associated with changes in histone modifications, with transcriptional repressive marks increased and enhancing marks decreased in closed chromatin regions, and vice versa in open regions [68]. Therefore, how DNA methylation variation at this region interplays with these changes in transcriptional regulation merits further investigation.

The next stage of our analysis focussed on identifying co-methylated networks associated with HD. We identified six modules showing a nominally significant ME difference between HD and control in the striatum, with the red module having a BH-corrected *Q* < 0.1. We did not identify any modules showing a significant ME difference in the entorhinal cortex or cerebellum. The four nominally significant striatum modules that also showed a significant and moderate correlation of MM and PS contained CpG sites annotated to genomic regions previously associated with HD from GWAS [15], although these did not pass multiple testing correction. Nonetheless, the green module contained probes annotated to the DNA mismatch repair genes, *MSH3* and *LIG1*. *MSH3* and *LIG1* are key genetic modifiers of age of motor symptom onset and the age to reach a Total Functional Capacity scale score of 6 (TFC6) out of 13, indicating a severe reduction in the capacity of an individual to perform daily tasks (a score of 13 represents full capacity) [69], whilst *MSH3* has also been identified as a genetic modifier of somatic expansion in HD blood [15, 66]. Probes annotated to *MSH3* were also part of the red and yellow modules, whilst probes annotated to two other modifiers of age of motor onset and age to reach TFC6; *RRM2B* (yellow) and *TCERG1* (green), were also present in the modules. In addition, we also observed three probes in the green module, two probes in the red module and two in the yellow module that were annotated to the *HTT* gene. Whilst the module CpGs were not significantly enriched at individual HD associated loci, the observations may indicate that differential regulation of DNA methylation is a feature of HD at genes that modify the disease course, and in the case of *HTT*, that house the disease-causing mutation.

Our co-methylation network analysis also supports previous methylomic associations with HD detected in blood [25]. Lu and colleagues reported three loci to be associated with HD motor progression, which were annotated to the genes *GRIK4*, *PEX14*, and *COX412* [25]. Unfortunately, our study was cross-sectional and did not have any suitable clinical data to explore associations with motor progression however, CpGs annotated to each of these genes were seen in our striatal HD associated modules. *GRIK4* was the most represented with six annotated CpGs in the yellow module, including two hub probes, and two CpGs in the green module, with one being a hub probe. *PEX14* had a probe feature in each of the red and yellow modules, with the latter narrowly missing our hub probe definition. *COX412* had a single annotated hub probe in the green module. Together this suggests similarities between HD associated methylation changes in the striatum and in blood, and further study of motor progression and DNA methylation in matched blood and striatal tissue may provide further mechanistic insights, as well as potential targets for therapeutic intervention to slow motor progression, or biomarkers to track symptomatic landmarks. GO and KEGG pathway analysis of the modules associated with HD revealed terms relating to several key cell signalling pathways. Manipulation of several of these pathways can alter outcomes in model systems of HD, indicating that differential methylation associated with HD in the striatum may play a role in mediating some of these effects. Inhibiting mTOR reduced neurodegeneration in a HD fly model and alleviated behavioral and motor phenotypes in mice [70]. Inhibition of TNFα in the R6/2 mouse model of HD partially rescued a reduction in brain weight [71], whilst GnRH is reduced in the same model, although increasing GnRH levels did not improve phenotypes [72]. A systematic review of huntingtin interacting proteins, which performed a clustering analysis and subsequent KEGG pathway analysis, also identified the Rap1 signalling pathway [73], which reached nominal significance in both our red and yellow modules. Finally, insulin treatment of human HD lymphoblasts has been shown to increase phosphorylation of Htt, which rescued energy metabolism in the cells [74]. Together these findings indicate key signalling pathways in HD are enriched in DNA methylation networks associated with HD.

We also observed terms that may provide insight into phenotypic behaviour in HD and how it relates to DNA methylation variation. In the green module our top KEGG pathway term related to morphine addiction, passing our most stringent BH threshold, Alcoholism was also nominally significant in the green and lavenderblush3 modules, whilst alcoholic liver disease passed the BH threshold in the yellow module and cellular response to alcohol was the top GO term in this module. Other addiction terms were also highlighted (yellow: amphetamine, lavenderblush3: cocaine, morphine). Addiction has long been anecdotally associated with HD; however, research has now begun to substantiate this association. Early epidemiological work has associated patient groups with the largest levels of alcohol consumption, having the largest increase in severity of psychiatric symptoms [75]. More recent studies have found that both alcohol and substance abuse is associated with a decreased age of motor symptom onset, and this association is stronger in women [76, 77]. Alcohol consumption per week has recently been associated with D2 SPNs in a study that utilized GWAS data of alcohol consumption and a large snRNA-seq human brain cell atlas dataset to map genomic variation to specific cell types [78]. Previous work has associated these neurons as contributing to alcohol consumption in a causative manner, with selective depletion of D2 dopamine receptors on iSPNs in mice resulting in increased sensitivity to alcohol and an increase in consumption [79]. Given that D2 SPNs are affected earliest in HD and to a larger degree than D1 SPNs [80], and as alcohol consumption is linked to early onset and worsened symptoms in the disease, enrichment of genes annotated to our HD associated co-methylation networks in this cell type may provide a link to understanding the biological mechanisms underlying this phenomenon. However, establishing a causal link would require integration of far larger and deeper phenotyped EWAS and genetic studies.

Terms relating to metabolic processes were also identified in our analysis, further strengthening links between our findings and pathological processes in HD. Purine metabolism and signalling were highlighted in the red, green and lavenderblush3 modules, whilst terms relating to nucleoside/nucleotide metabolism featured in the red and green module hub probes. Perturbation in cellular metabolic processes have long been known to result from the *HTT* mutation [81], and it is known that metabolic changes can impact on DNA methylation, through alterations to cellular concentrations of the methyl donor *S*-adenosylmethionine (SAM) [82]. We therefore compared global methylation levels between the HD and control groups in each brain region, but saw no difference. This suggests that the loci-specific HD-associated changes we observed in the striatum were not solely the result of metabolic alterations. HD-associated metabolomic alterations have previously been associated with white matter loss in a study of pre-manifest HD patients [83]. Through matching imaged regions to gene expression array data generated in the same regions, the authors observed that in longitudinal analysis of corticostriatal white matter loss, there was an association with metabolism related GO terms, whilst cross-sectional analysis showed an association with synaptic terms, matching the associations we observed, particularly in our green module. Future work should further clarify the links between disrupted metabolic processes and DNA methylation in HD through metabolomic and DNA methylation profiling in the same samples.

Alterations in metabolic activity have been observed in iPSC derived astrocytes with polyglutamine expansions, with shorter pathogenic repeat cells exhibiting increased activity whilst astrocytes with longer repeats had decreases in metabolism [84]. In the current study, we observed FDR significant enrichment of astrocyte-expressed genes in our yellow module and of SPN-expressed genes in our red and green modules. A primary limitation of our study, however, is the use of DNA derived from bulk brain tissue. Pertinent to this, it has been reported that key marker genes in the cell types of the striatum show altered transcriptional profiles in HD. Modules of genes associated with SPNs were observed to be downregulated in these cell types but upregulated in glial cells including astrocytes and oligodendrocytes, whilst glial associated gene modules saw the reverse, with downregulation of the respective modules in astrocytes and oligodendrocytes, but upregulation in SPNs [85]. Therefore, if cells in the striatum lose a sense of clear transcriptional identity in HD, bulk tissue analysis would not be able to capture which cell types are driving the association between DNA co-methylation networks and particular pathways. It is believed that astrocytes may support axon development [86], and as we observed significant terms related to axon development in our yellow module, it may mean we have captured a true astrocyte-enriched network. However, given that DNA methylation differs from cell-type to cell-type, this highlights the importance of profiling isolated cell populations in future studies. Indeed, DNA methylation profiling on the EPIC array has been undertaken on fluorescence activated nuclei sorted (FANS) cell populations in AD [38], and so future studies should also employ FANS to explore cell-type specific DNA methylation changes in HD.

FANS would also allow the identification of the cell type responsible for one of our strongest findings, namely hypomethylation in the *PTGDS* gene in the striatum. Whilst *PTGDS* is expressed by oligodendrocytes, it has higher expression in oligodendrocyte progenitor cells and expression levels decrease as the cells differentiate [87]. Furthermore, *PTGDS* expression has been shown to be negatively correlated with CAG length in oligodendrocyte progenitor cells and oligodendrocytes isolated from HD striatum, suggesting that oligodendrocyte maturation is dysregulated in HD [63]. Given that disrupted myelination is a feature of HD mouse models [88], and as *PTGDS* gene expression is increased in white matter in multiple sclerosis, which is another disease that affects myelination [89], the relationship between *PTGDS* gene regulation within specific populations of glia and its impact on disease requires further examination.

Another caveat of the current study is the limited phenotypic and clinical information available in the HD cohort. Pathological staging was not available for most of the cohort and given the importance of disease stage on symptom severity [8] and cellular changes in the brain [90], heterogeneity in the HD cohort in terms of disease stage could introduce variation. This is particularly pertinent given that epigenetic age acceleration in HD may not increase linearly with pathological stage [23]. Similarly, although we profiled the striatum, the specific anatomical subdivision was not known. Given that there is known variation in neurodegeneration across the divisions of the striatum, this may also introduce further heterogeneity into our HD cohort [50]. Finally, there was no information on age of symptom onset, or CAG repeat length available from the Brain Banks, meaning that we could not explore methylation signatures associated with these clinical variables at the current time, and thus leaving us unable to confirm previous associations of DNA methylation with age of onset [9, 24]. Future studies should be undertaken in well phenotyped cohorts with detailed clinical and pathological characterization. Although the Illumina EPIC array is a cost-effective platform for high-throughput assessment of DNA methylation, it only assesses approximately 850,000 CpG sites, meaning that it is not truly assessing the whole genome, and we may have missed some important HD-associated methylation. Looking to the future other technologies, such as bisulfite short-read DNA sequencing or long-read DNA sequencing will give a better understanding of the DNA methylation landscape in HD, enabling further interrogation of the highly correlated region within the *PTGDS* gene. In addition, long-read platforms have the added advantage of being able to simultaneously profile genetic variation (e.g., CAG repeat length) and DNA methylation [91] in the same sample. This would enable detailed interrogation on the relationship between CAG repeat length, DNA methylation at the *HTT* gene, as well as other genes associated with age of onset of HD. This is particularly pertinent as our enrichment analysis of DNA methylation of the *HTT* genomic region showed that it narrowly missed nominal significance, which could be due to the power of our study, or the limited number of CpGs in this region covered by the array we used. Finally, the focus of this study has been on DNA methylation however, a number of different epigenetic processes work together to fine tune genomic function. It will be of considerable interest to profile additional epigenetic marks and integrate this with gene transcription in the future.

Given the current work strengthens associations between HD and physiological disturbances to the urea cycle and metabolism, it suggests these systems may prove pertinent targets for therapeutic intervention. Similarly, concordance between annotated genes associated with HD motor progression in blood and with loci that featured in our HD-associated co-methylated networks, suggest that further study into the association of DNA methylation and phenotypic variation in HD, particularly in matched striatal and blood samples from the same individual, may highlight biomarkers that could track the development of the disease course, aiding the design of interventions to target specific therapeutic windows.

## Conclusions

The current study provides further evidence that differential DNA methylation is a feature of HD, particularly in the striatum. Although no robust significant changes were detected in the entorhinal cortex, comparisons between the striatal and entorhinal cortex datasets indicate that better powered studies may uncover associations in this region. Encouragingly, several of the loci were identified in genes that have had previous association with HD. We also identified modules of HD-associated co-methylation networks associated with pathways with biological relevance to disease and that were enriched in disease relevant cell types. We nominate a novel finding of robust differential methylation at *PTGDS,* a gene with transcriptional alterations in oligodendrocytes in HD. These findings suggest that the current study has found novel alterations in epigenetic regulation at key genes and that their association with HD requires further research.

## Supplementary Information

Below is the link to the electronic supplementary material.

Supplementary Material 1

Supplementary Material 2

## Data Availability

The datasets generated and analyzed during the current study are available in the Gene Expression Omnibus (GEO) repository (GSE297210). Analytical scripts used in this manuscript are available at https://github.com/UoE-Dementia-Genomics/HD-DNAmeth.

## Abbreviations

450K: Illumina Infinium 450K methylation array
5mC: 5-methylcytosine
Aβ: Amyloid beta
AD: Alzheimer’s disease
*ADORA2A*: Adenosine A_2A_ receptor
BH: Benjamini–Hochberg
bp: Base pairs
CBB: Cambridge brain bank
D1: Dopamine receptor D1 expressing
D2: Dopamine receptor D2 expressing
DMP: Differentially methylated position
DMR: Differentially methylated region
dSPN: Direct pathway spiny projection neuron
EPIC: Illumina infinium EPIC methylation array
eQTL: Expression quantitative trait loci
ES: Effect size
EWAS: Epigenome-wide association study
EWCE: Expression weighted cell type enrichment
FANS: Fluorescence activated nuclei sorting
FDR: False discovery rate
*FOXP2*: *FOXP2/OLFM3*-Expressing striatal neurons
GO: Gene ontology
GEO: Gene expression omnibus
GnRH: Gonadotropin-releasing hormone
GWAS: Genome-wide association study
HD: Huntington’s disease
iPSC: Induced pluripotent stem cell
iSPN: Indirect pathway spiny projection neuron
KEGG: Kyoto encyclopaedia of genes and genomes
LNDBB: London neurodegenerative diseases brain bank
MAF: Minor allele frequency
MBB: Manchester brain bank
ME: Module eigengene
MM: Module membership
NeuN: Neuronal nuclear protein
OBB: Oxford brain bank
*P*: P-value
PC: Principal component
PCA: Principal component analysis
PS: Probe significance
*Q*: FDR (BH) corrected *P*-value (*Q*-value)
QC: Quality control
QQ: Quantile–quantile
qRT-PCR: Quantitative real-time polymerase chain reaction
*r*: Pearson’s coefficient
SD: Standard deviation
SE: Summarized experiment
SNP: Single nucleotide polymorphism
snRNA-seq: Single nuclei RNA sequencing
SPN: Spiny projection neuron
TFC6: Total functional capacity scale score of 6
TNF: Tumour necrosis factor
TSS: Transcriptional start site
WGCNA: Weighted gene correlation network analysis

## References

[1] MacDonald ME, Ambrose CM, Duyao MP, Myers RH, Lin C, Srinidhi L, et al. A novel gene containing a trinucleotide repeat that is expanded and unstable on Huntington’s disease chromosomes. Cell. 1993;72(6):971–83. 8458085 10.1016/0092-8674(93)90585-e

[2] Novak MJ, Tabrizi SJ. Huntington’s disease. BMJ. 2010. 10.1136/bmj.c3109. 20591965 10.1136/bmj.c3109

[3] Poudel GR, Harding IH, Egan GF, Georgiou‐Karistianis N. Network spread determines severity of degeneration and disconnection in Huntington’s disease. Hum Brain Mapp. 2019;40(14):4192–201. 31187915 10.1002/hbm.24695 PMC6865500

[4] Albin RL, Reiner A, Anderson KD, Dure LS IV, Handelin B, Balfour R, et al. Preferential loss of striato-external pallidal projection neurons in presymptomatic Huntington’s disease. Ann Neurol. 1992;31(4):425–30. 1375014 10.1002/ana.410310412

[5] Rosas HD, Salat DH, Lee SY, Zaleta AK, Pappu V, Fischl B, et al. Cerebral cortex and the clinical expression of Huntington’s disease: complexity and heterogeneity. Brain. 2008;131(4):1057–68. 18337273 10.1093/brain/awn025 PMC2657201

[6] Braak H, Braak E. Allocortical involvement in Huntington’s disease. Neuropathol Appl Neurobiol. 1992;18(6):539–47. 1488086 10.1111/j.1365-2990.1992.tb00824.x

[7] Singh‐Bains MK, Mehrabi NF, Sehji T, Austria MD, Tan AY, Tippett LJ, et al. Cerebellar degeneration correlates with motor symptoms in Huntington disease. Ann Neurol. 2019;85(3):396–405. 30635944 10.1002/ana.25413 PMC6590792

[8] Tabrizi SJ, Scahill RI, Durr A, Roos RA, Leavitt BR, Jones R, et al. Biological and clinical changes in premanifest and early stage Huntington’s disease in the TRACK-HD study: the 12-month longitudinal analysis. Lancet Neurol. 2011;10(1):31–42. 21130037 10.1016/S1474-4422(10)70276-3

[9] Duyao M, Ambrose C, Myers R, Novelletto A, Persichetti F, Frontali M, et al. Trinucleotide repeat length instability and age of onset in Huntington’s disease. Nat Genet. 1993;4(4):387–92. 8401587 10.1038/ng0893-387

[10] Langbehn DR, Brinkman RR, Falush D, Paulsen JS, Hayden M, Group aIHsDC. A new model for prediction of the age of onset and penetrance for Huntington’s disease based on CAG length. Clin Genet. 2004;65(4):267–77. 15025718 10.1111/j.1399-0004.2004.00241.x

[11] Project* UVCR, Wexler NS. Venezuelan kindreds reveal that genetic and environmental factors modulate Huntington’s disease age of onset. Proc Natl Acad Sci U S A. 2004;101(10):3498–503. 14993615 10.1073/pnas.0308679101 PMC373491

[12] Keum JW, Shin A, Gillis T, Mysore JS, Elneel KA, Lucente D, et al. The HTT CAG-expansion mutation determines age at death but not disease duration in Huntington disease. Am J Hum Genet. 2016;98(2):287–98. 26849111 10.1016/j.ajhg.2015.12.018 PMC4746370

[13] Lee J-M, Wheeler VC, Chao MJ, Vonsattel JPG, Pinto RM, Lucente D, et al. Identification of genetic factors that modify clinical onset of Huntington’s disease. Cell. 2015;162(3):516–26. 26232222 10.1016/j.cell.2015.07.003 PMC4524551

[14] Flower M, Lomeikaite V, Ciosi M, Cumming S, Morales F, Lo K, et al. MSH3 modifies somatic instability and disease severity in Huntington’s and myotonic dystrophy type 1. Brain. 2019;142(7):1876–86. 31216018 10.1093/brain/awz115 PMC6598626

[15] Lee J-M, Correia K, Loupe J, Kim K-H, Barker D, Hong EP, et al. CAG repeat not polyglutamine length determines timing of Huntington’s disease onset. Cell. 2019;178(4):887-900.e14. 31398342 10.1016/j.cell.2019.06.036 PMC6700281

[16] Roubroeks JAY, Smith RG, van den Hove DLA, Lunnon K. Epigenetics and DNA methylomic profiling in Alzheimer’s disease and other neurodegenerative diseases. J Neurochem. 2017;143(2):158–70. 28805248 10.1111/jnc.14148

[17] Smith AR, Wheildon G, Lunnon K. Invited review–a 5‐year update on epigenome‐wide association studies of DNA modifications in Alzheimer’s disease: progress, practicalities and promise. Neuropathol Appl Neurobiol. 2020;46(7):641–53. 32744362 10.1111/nan.12650

[18] Weymouth L, Smith AR, Lunnon K. DNA methylation in Alzheimer’s disease. 2024. 10.1007/7854_2024_530 39455499

[19] MacBean LF, Smith AR, Lunnon K. Exploring beyond the DNA sequence: a review of epigenomic studies of DNA and histone modifications in dementia. Curr Genet Med Rep. 2020;8:79–92.

[20] Harvey J, Pishva E, Chouliaras L, Lunnon K. Elucidating distinct molecular signatures of Lewy body dementias. Neurobiol Dis. 2023;188:106337. 37918758 10.1016/j.nbd.2023.106337

[21] Harvey J, Imm J, Kouhsar M, Smith AR, Creese B, Smith RG, et al. Interrogating DNA methylation associated with Lewy body pathology in a cross brain-region and multi-cohort study. medRxiv. 2025

[22] Ng CW, Yildirim F, Yap YS, Dalin S, Matthews BJ, Velez PJ, et al. Extensive changes in DNA methylation are associated with expression of mutant huntingtin. Proc Natl Acad Sci. 2013;110(6):2354–9. 23341638 10.1073/pnas.1221292110 PMC3568325

[23] Horvath S, Langfelder P, Kwak S, Aaronson J, Rosinski J, Vogt TF, et al. Huntington’s disease accelerates epigenetic aging of human brain and disrupts DNA methylation levels. Aging. 2016;8(7):1485. 27479945 10.18632/aging.101005 PMC4993344

[24] De Souza RA, Islam SA, McEwen LM, Mathelier A, Hill A, Mah SM, et al. DNA methylation profiling in human Huntington’s disease brain. Hum Mol Genet. 2016;25(10):2013–30. 26953320 10.1093/hmg/ddw076

[25] Lu AT, Narayan P, Grant MJ, Langfelder P, Wang N, Kwak S, et al. DNA methylation study of Huntington’s disease and motor progression in patients and in animal models. Nat Commun. 2020;11(1):1–15. 32913184 10.1038/s41467-020-18255-5 PMC7484780

[26] Pircs K, Drouin-Ouellet J, Gil J, Rezeli M, Grassi DA, Garza R, et al. Distinct sub-cellular autophagy impairments occur independently of protein aggregation in induced neurons from patients with Huntington’s disease. bioRxiv. 2021.

[27] Smith AR, Smith RG, Burrage J, Troakes C, Al-Sarraj S, Kalaria RN, et al. A cross-brain regions study of ANK1 DNA methylation in different neurodegenerative diseases. Neurobiol Aging. 2019;74:70–6. 30439595 10.1016/j.neurobiolaging.2018.09.024

[28] Team RC. R: A language and environment for statistical computing: R Foundation for Statistical Computing, Vienna, 2018.

[29] Gentleman RC, Carey VJ, Bates DM, Bolstad B, Dettling M, Dudoit S, et al. Bioconductor: open software development for computational biology and bioinformatics. Genome Biol. 2004;5(10):R80. 15461798 10.1186/gb-2004-5-10-r80 PMC545600

[30] Davis S, Du P, Bilke S, Triche T, Bootwalla M. methylumi: Handle Illumina methylation data. R package version. 2015;2.

[31] Aryee MJ, Jaffe AE, Corrada-Bravo H, Ladd-Acosta C, Feinberg AP, Hansen KD, et al. Minfi: a flexible and comprehensive Bioconductor package for the analysis of Infinium DNA methylation microarrays. Bioinformatics. 2014;30(10):1363–9. 24478339 10.1093/bioinformatics/btu049 PMC4016708

[32] Pidsley R, Y Wong CC, Volta M, Lunnon K, Mill J, Schalkwyk LC. A data-driven approach to preprocessing Illumina 450K methylation array data. BMC Genomics. 2013;14(1):1–10. 23631413 10.1186/1471-2164-14-293 PMC3769145

[33] McCartney DL, Walker RM, Morris SW, McIntosh AM, Porteous DJ, Evans KL. Identification of polymorphic and off-target probe binding sites on the Illumina Infinium MethylationEPIC BeadChip. Genom Data. 2016;9:22–4. 27330998 10.1016/j.gdata.2016.05.012 PMC4909830

[34] Guintivano J, Aryee MJ, Kaminsky ZA. A cell epigenotype specific model for the correction of brain cellular heterogeneity bias and its application to age, brain region and major depression. Epigenetics. 2013;8(3):290–302. 23426267 10.4161/epi.23924 PMC3669121

[35] Gusel’Nikova V, Korzhevskiy D. NeuN as a neuronal nuclear antigen and neuron differentiation marker. Acta Naturae (aнглoязычнaя вepcия). 2015;7(2 (25)):42–7. PMC4463411 26085943

[36] van Iterson M, van Zwet EW, Heijmans BT. Controlling bias and inflation in epigenome- and transcriptome-wide association studies using the empirical null distribution. Genome Biol. 2017;18(1):19. 28129774 10.1186/s13059-016-1131-9 PMC5273857

[37] Lunnon K, Smith R, Hannon E, De Jager PL, Srivastava G, Volta M, et al. Methylomic profiling implicates cortical deregulation of ANK1 in Alzheimer’s disease. Nat Neurosci. 2014;17(9):1164–70. 25129077 10.1038/nn.3782 PMC4410018

[38] Shireby G, Dempster EL, Policicchio S, Smith RG, Pishva E, Chioza B, et al. DNA methylation signatures of Alzheimer’s disease neuropathology in the cortex are primarily driven by variation in non-neuronal cell-types. Nat Commun. 2022;13(1):5620. 36153390 10.1038/s41467-022-33394-7 PMC9509387

[39] Smith RG, Pishva E, Shireby G, Smith AR, Roubroeks JA, Hannon E, et al. A meta-analysis of epigenome-wide association studies in Alzheimer’s disease highlights novel differentially methylated loci across cortex. Nat Commun. 2021;12(1):1–13. 34112773 10.1038/s41467-021-23243-4 PMC8192929

[40] Pedersen BS, Schwartz DA, Yang IV, Kechris KJ. Comb-p: software for combining, analyzing, grouping and correcting spatially correlated P-values. Bioinformatics. 2012;28(22):2986–8. 22954632 10.1093/bioinformatics/bts545 PMC3496335

[41] Bellenguez C, Küçükali F, Jansen IE, Kleineidam L, Moreno-Grau S, Amin N, et al. New insights into the genetic etiology of Alzheimer’s disease and related dementias. Nat Genet. 2022;54(4):412–36. 35379992 10.1038/s41588-022-01024-z PMC9005347

[42] Langfelder P, Horvath S. WGCNA: an R package for weighted correlation network analysis. BMC Bioinform. 2008;9(1):559. 10.1186/1471-2105-9-559 PMC2631488 19114008

[43] Phipson B, Maksimovic J, Oshlack A. missMethyl: an R package for analyzing data from Illumina’s HumanMethylation450 platform. Bioinformatics. 2016;32(2):286–8. 26424855 10.1093/bioinformatics/btv560

[44] Sayols S. rrvgo: a Bioconductor package for interpreting lists of Gene Ontology terms. MicroPubl Biol. 2023;2023. 10.17912/micropub.biology.000811 PMC10155054 37151216

[45] Lee H, Fenster RJ, Pineda SS, Gibbs WS, Mohammadi S, Davila-Velderrain J, et al. Cell type-specific transcriptomics reveals that mutant Huntingtin leads to mitochondrial RNA release and neuronal innate immune activation. Neuron. 2020;107(5):891-908.e8. 32681824 10.1016/j.neuron.2020.06.021 PMC7486278

[46] Skene NG, Grant SG. Identification of vulnerable cell types in major brain disorders using single cell transcriptomes and expression weighted cell type enrichment. Front Neurosci. 2016;10:179460. 10.3389/fnins.2016.00016 PMC4730103 26858593

[47] Peters J, Williamson CM. Control of imprinting at the Gnas cluster. Epigenetics. 2007;2(4):207–13. 18094621 10.4161/epi.2.4.5380

[48] Kosaki K, Kosaki R, Craigen WJ, Matsuo N. Isoform-specific imprinting of the human PEG1/MEST gene. Am J Hum Genet. 2000;66(1):309–12. 10631159 10.1086/302712 PMC1288335

[49] Märtin A, Calvigioni D, Tzortzi O, Fuzik J, Wärnberg E, Meletis K. A spatiomolecular map of the striatum. Cell Rep. 2019;29(13):4320-33. e5. 31875543 10.1016/j.celrep.2019.11.096

[50] Vonsattel JPG, Keller C, Ramirez EPC. Huntington’s disease–neuropathology. Handb Clin Neurol. 2011;100:83–100. 21496571 10.1016/B978-0-444-52014-2.00004-5

[51] Villar-Menéndez I, Blanch M, Tyebji S, Pereira-Veiga T, Albasanz JL, Martín M, et al. Increased 5-methylcytosine and decreased 5-hydroxymethylcytosine levels are associated with reduced striatal A2AR levels in Huntington’s disease. Neuromol Med. 2013;15(2):295–309. 10.1007/s12017-013-8219-0 23385980

[52] Lin YH, Zhen YY, Chien KY, Lee IC, Lin WC, Chen MY, et al. LIMCH1 regulates nonmuscle myosin-II activity and suppresses cell migration. Mol Biol Cell. 2017;28(8):1054–65. 28228547 10.1091/mbc.E15-04-0218 PMC5391182

[53] García D, Ordenes P, Benítez J, González A, García-Robles MA, López V, et al. Cloning of two LIMCH1 isoforms: characterization of their distribution in rat brain and their agmatinase activity. Histochem Cell Biol. 2016;145:305–13. 26678503 10.1007/s00418-015-1389-0

[54] Patassini S, Begley P, Xu J, Church SJ, Reid SJ, Kim EH, et al. Metabolite mapping reveals severe widespread perturbation of multiple metabolic processes in Huntington’s disease human brain. Biochem Biophys Acta. 2016;1862(9):1650–62. 27267344 10.1016/j.bbadis.2016.06.002

[55] Bichell TJV, Wegrzynowicz M, Tipps KG, Bradley EM, Uhouse MA, Bryan M, et al. Reduced bioavailable manganese causes striatal urea cycle pathology in Huntington’s disease mouse model. Biochem Biophys Acta. 2017;1863(6):1596–604. 10.1016/j.bbadis.2017.02.013 PMC5515276 28213125

[56] Lee Hyun K, Deneen B. Daam2 is required for dorsal patterning via modulation of canonical Wnt signaling in the developing spinal cord. Dev Cell. 2012;22(1):183–96. 22227309 10.1016/j.devcel.2011.10.025 PMC3264735

[57] Strand AD, Aragaki AK, Shaw D, Bird T, Holton J, Turner C, et al. Gene expression in Huntington’s disease skeletal muscle: a potential biomarker. Hum Mol Genet. 2005;14(13):1863–76. 15888475 10.1093/hmg/ddi192

[58] Nishimura T, Kubosaki A, Ito Y, Notkins AL. Disturbances in the secretion of neurotransmitters in IA-2/IA-2β null mice: changes in behavior, learning and lifespan. Neuroscience. 2009;159(2):427–37. 19361477 10.1016/j.neuroscience.2009.01.022 PMC2672562

[59] Nishimura T, Harashima S-i, Yafang H, Notkins AL. IA-2 modulates dopamine secretion in PC12 cells. Mol Cell Endocrinol. 2010;315(1):81–6. 19799965 10.1016/j.mce.2009.09.023 PMC3495171

[60] The Hd iPsc Consortium. Induced pluripotent stem cells from patients with Huntington’s disease Show CAG-repeat-expansion-associated phenotypes. Cell Stem Cell. 2012;11(2):264–78. 22748968 10.1016/j.stem.2012.04.027 PMC3804072

[61] Skotte NH, Andersen JV, Santos A, Aldana BI, Willert CW, Nørremølle A, et al. Integrative characterization of the R6/2 mouse model of Huntington’s disease reveals dysfunctional astrocyte metabolism. Cell Rep. 2018;23(7):2211–24. 29768217 10.1016/j.celrep.2018.04.052

[62] Kume S, Lee Y-H, Miyamoto Y, Fukada H, Goto Y, Inui T. Systematic interaction analysis of human lipocalin-type prostaglandin D synthase with small lipophilic ligands. Biochem J. 2012;446(2):279–89. 22677050 10.1042/BJ20120324

[63] Lim RG, Al-Dalahmah O, Wu J, Gold MP, Reidling JC, Tang G, et al. Huntington disease oligodendrocyte maturation deficits revealed by single-nucleus RNAseq are rescued by thiamine-biotin supplementation. Nat Commun. 2022;13(1):7791. 36543778 10.1038/s41467-022-35388-x PMC9772349

[64] Kanekiyo T, Ban T, Aritake K, Huang Z-L, Qu W-M, Okazaki I, et al. Lipocalin-type prostaglandin D synthase/β-trace is a major amyloid β-chaperone in human cerebrospinal fluid. Proc Natl Acad Sci. 2007;104(15):6412–7. 17404210 10.1073/pnas.0701585104 PMC1851035

[65] Handley RR, Reid SJ, Brauning R, Maclean P, Mears ER, Fourie I, et al. Brain urea increase is an early Huntington’s disease pathogenic event observed in a prodromal transgenic sheep model and HD cases. Proc Natl Acad Sci USA. 2017;114(52):E11293–302. 29229845 10.1073/pnas.1711243115 PMC5748180

[66] Consortium GMoHsD, Lee J-M, McLean ZL, Correia K, Shin JW, Lee S, et al. Genetic modifiers of somatic expansion and clinical phenotypes in Huntington's disease reveal shared and tissue-specific effects. bioRxiv. 2024:2024.06. 10.597797. 10.1038/s41588-025-02191-5 PMC13132264 40490511

[67] Võsa U, Claringbould A, Westra H-J, Bonder MJ, Deelen P, Zeng B, et al. Large-scale cis- and trans-eQTL analyses identify thousands of genetic loci and polygenic scores that regulate blood gene expression. Nat Genet. 2021;53(9):1300–10. 34475573 10.1038/s41588-021-00913-z PMC8432599

[68] Alcalá-Vida R, Seguin J, Lotz C, Molitor AM, Irastorza-Azcarate I, Awada A, et al. Age-related and disease locus-specific mechanisms contribute to early remodelling of chromatin structure in Huntington’s disease mice. Nat Commun. 2021;12(1):364. 33441541 10.1038/s41467-020-20605-2 PMC7807045

[69] Lee J-M, Huang Y, Orth M, Gillis T, Siciliano J, Hong E, et al. Genetic modifiers of Huntington disease differentially influence motor and cognitive domains. Am J Hum Genet. 2022;109(5):885–99. 35325614 10.1016/j.ajhg.2022.03.004 PMC9118113

[70] Ravikumar B, Vacher C, Berger Z, Davies JE, Luo S, Oroz LG, et al. Inhibition of mTOR induces autophagy and reduces toxicity of polyglutamine expansions in fly and mouse models of Huntington disease. Nat Genet. 2004;36(6):585–95. 15146184 10.1038/ng1362

[71] Pido-Lopez J, Tanudjojo B, Farag S, Bondulich M-K, Andre R, Tabrizi SJ, et al. Inhibition of tumour necrosis factor alpha in the R6/2 mouse model of Huntington’s disease by etanercept treatment. Sci Rep. 2019;9(1):7202. 31076648 10.1038/s41598-019-43627-3 PMC6510744

[72] Papalexi E, Persson A, Björkqvist M, Petersén Å, Woodman B, Bates GP, et al. Reduction of GnRH and infertility in the R6/2 mouse model of Huntington’s disease. Eur J Neurosci. 2005;22(6):1541–6. 16190907 10.1111/j.1460-9568.2005.04324.x

[73] Podvin S, Rosenthal SB, Poon W, Wei E, Fisch KM, Hook V. Mutant Huntingtin protein interaction map implicates dysregulation of multiple cellular pathways in neurodegeneration of Huntington’s Disease. J Huntingtons Dis. 2022;11:243–67. 35871359 10.3233/JHD-220538 PMC9484122

[74] Naia L, Ferreira IL, Cunha-Oliveira T, Duarte AI, Ribeiro M, Rosenstock TR, et al. Activation of IGF-1 and insulin signaling pathways ameliorate mitochondrial function and energy metabolism in Huntington’s Disease human lymphoblasts. Mol Neurobiol. 2015;51:331–48. 24841383 10.1007/s12035-014-8735-4

[75] Ehret JC, Day PS, Wiegand R, Wojcieszek J, Chambers RA. Huntington disease as a dual diagnosis disorder: data from the national research roster for Huntington disease patients and families. Drug Alcohol Depend. 2007;86(2):283–6. 16930866 10.1016/j.drugalcdep.2006.06.009 PMC2877623

[76] Byars JA, Beglinger LJ, Moser DJ, Gonzalez-Alegre P, Nopoulos P. Substance abuse may be a risk factor for earlier onset of Huntington disease. J Neurol. 2012;259:1824–31. 22274789 10.1007/s00415-012-6415-8

[77] Schultz JL, Kamholz JA, Moser DJ, Feely SME, Paulsen JS, Nopoulos PC. Substance abuse may hasten motor onset of Huntington disease. Neurology. 2017;88(9):909–15. 28148631 10.1212/WNL.0000000000003661 PMC5331869

[78] Duncan LE, Li T, Salem M, Li W, Mortazavi L, Senturk H, et al. Mapping the cellular etiology of schizophrenia and complex brain phenotypes. Nat Neurosci. 2025;28(2):248–58. 39833308 10.1038/s41593-024-01834-w PMC11802450

[79] Bocarsly ME, da Silva e Silva D, Kolb V, Luderman KD, Shashikiran S, Rubinstein M, et al. A Mechanism linking two known vulnerability factors for alcohol abuse: heightened alcohol stimulation and low striatal dopamine D2 receptors. Cell Rep. 2019;29(5):1147–63. 31665630 10.1016/j.celrep.2019.09.059 PMC6880649

[80] Han I, You Y, Kordower JH, Brady ST, Morfini GA. Differential vulnerability of neurons in Huntington’s disease: the role of cell type-specific features. J Neurochem. 2010;113(5):1073–91. 20236390 10.1111/j.1471-4159.2010.06672.x PMC2890032

[81] Lee J-M, Ivanova EV, Seong IS, Cashorali T, Kohane I, Gusella JF, et al. Unbiased gene expression analysis implicates the huntingtin polyglutamine tract in extra-mitochondrial energy metabolism. PLoS Genet. 2007;3(8):e135. 17708681 10.1371/journal.pgen.0030135 PMC1950164

[82] Ulrey CL, Liu L, Andrews LG, Tollefsbol TO. The impact of metabolism on DNA methylation. Hum Mol Genet. 2005;14(suppl_1):R139–47. 15809266 10.1093/hmg/ddi100

[83] McColgan P, Gregory S, Seunarine KK, Razi A, Papoutsi M, Johnson E, et al. Brain regions showing white matter loss in Huntington’s disease are enriched for synaptic and metabolic genes. Biol Psychiatry. 2018;83(5):456–65. 29174593 10.1016/j.biopsych.2017.10.019 PMC5803509

[84] Lange J, Gillham O, Flower M, Ging H, Eaton S, Kapadia S, et al. PolyQ length-dependent metabolic alterations and DNA damage drive human astrocyte dysfunction in Huntington’s disease. Prog Neurobiol. 2023;225:102448. 37023937 10.1016/j.pneurobio.2023.102448

[85] Malaiya S, Cortes-Gutierrez M, Herb BR, Coffey SR, Legg SR, Cantle JP, et al. Single-nucleus RNA-seq reveals dysregulation of striatal cell identity due to huntington’s disease mutations. J Neurosci. 2021;41(25):5534–52. 34011527 10.1523/JNEUROSCI.2074-20.2021 PMC8221598

[86] Reemst K, Noctor SC, Lucassen PJ, Hol EM. The indispensable roles of microglia and astrocytes during brain development. Front Hum Neurosci. 2016;10:566. 27877121 10.3389/fnhum.2016.00566 PMC5099170

[87] Sakry D, Yigit H, Dimou L, Trotter J. Oligodendrocyte precursor cells synthesize neuromodulatory factors. PLoS ONE. 2015;10(5):e0127222. 25966014 10.1371/journal.pone.0127222 PMC4429067

[88] Ferrari Bardile C, Garcia-Miralles M, Caron NS, Rayan NA, Langley SR, Harmston N, et al. Intrinsic mutant HTT-mediated defects in oligodendroglia cause myelination deficits and behavioral abnormalities in Huntington disease. Proc Natl Acad Sci USA. 2019;116(19):9622–7. 31015293 10.1073/pnas.1818042116 PMC6511031

[89] Kagitani-Shimono K, Mohri I, Oda H, Ozono K, Suzuki K, Urade Y, et al. Lipocalin-type prostaglandin D synthase (β-trace) is upregulated in the αB-crystallin-positive oligodendrocytes and astrocytes in the chronic multiple sclerosis. Neuropathol Appl Neurobiol. 2006;32(1):64–73. 16409554 10.1111/j.1365-2990.2005.00690.x

[90] Vonsattel J-P, Myers RH, Stevens TJ, Ferrante RJ, Bird ED, Richardson JEP. Neuropathological classification of Huntington’s disease. J Neuropathol Exp Neurol. 1985;44(6):559–77. 2932539 10.1097/00005072-198511000-00003

[91] Rand AC, Jain M, Eizenga JM, Musselman-Brown A, Olsen HE, Akeson M, et al. Mapping DNA methylation with high-throughput nanopore sequencing. Nat Methods. 2017;14(4):411–3. 28218897 10.1038/nmeth.4189 PMC5704956

