# Supplementary material for: DNA methylation profiling in Huntington’s disease reveals disease associated changes in the striatum
Source: Clin Epigenetics. 2026 May 26;18:92. doi: 10.1186/s13148-026-02082-4 (PMC13202909; doi:10.1186/s13148-026-02082-4)
Supplement: Supplementary file 1 — Supplementary Material 1 [file 13148_2026_2082_MOESM1_ESM.pdf]

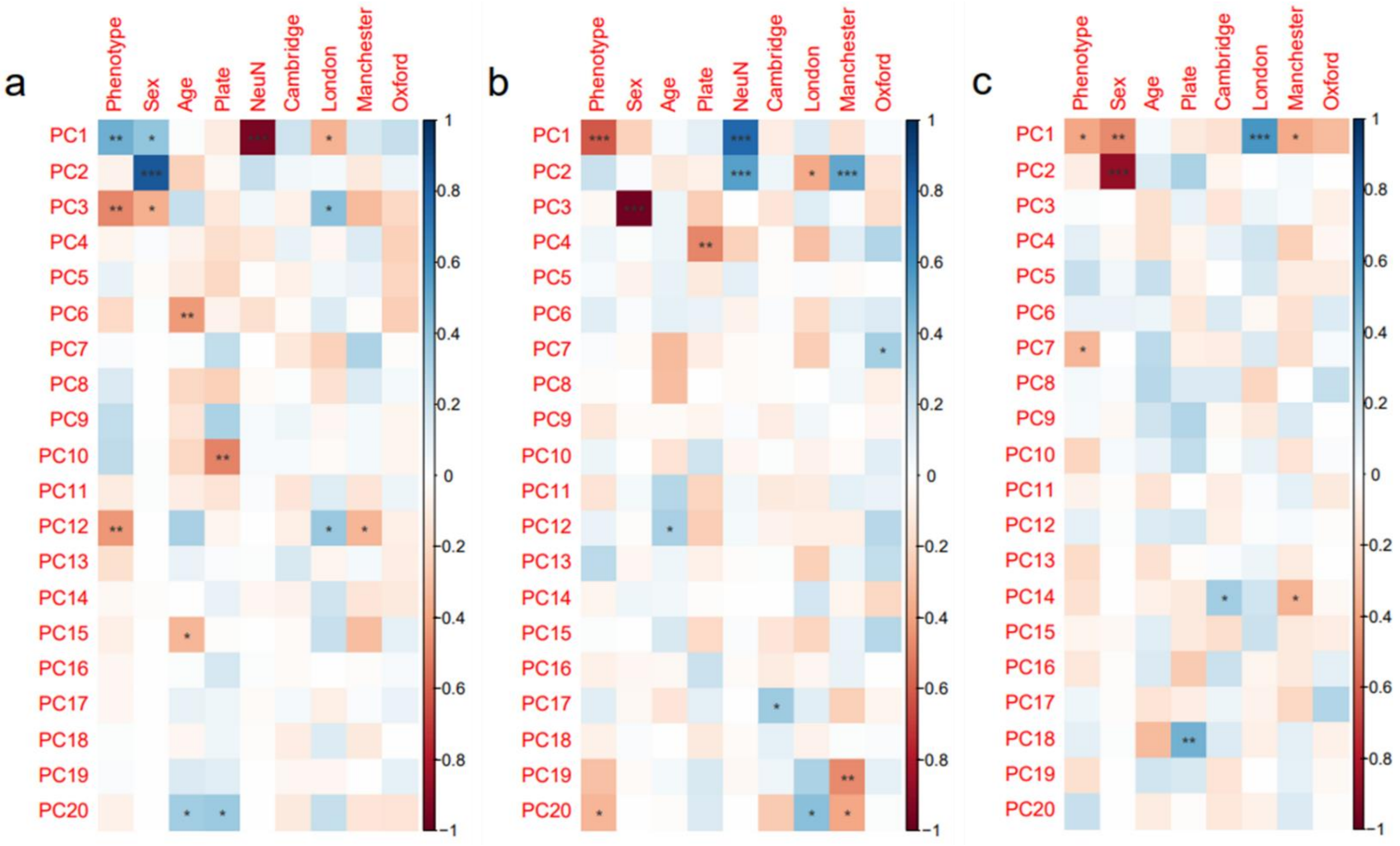

Supplementary Figure 1: Correlation heatmaps depicting correlations between each of the first 20 principal components (PCs) and selected covariates. Shown are heatmap correlations for (**a**), the striatum, (**b**), the entorhinal cortex, (**c**), the cerebellum. Deepening blue squares represent a stronger positive correlation, whilst darker red squares represent a stronger negative correlation. The significance level of the correlations is denoted by the *P*-value, which is represented as *** ($P < 0.001$), ** ($P < 0.01$) and * ($P < 0.05$). Key: Phenotype denotes disease (HD) status, Plate represents bisulfite conversion plate, NeuN represents neuronal cell proportion, Cambridge, London, Manchester, and Oxford represent the brain banks: CBB, LNDBB, MBB and OBB, respectively.

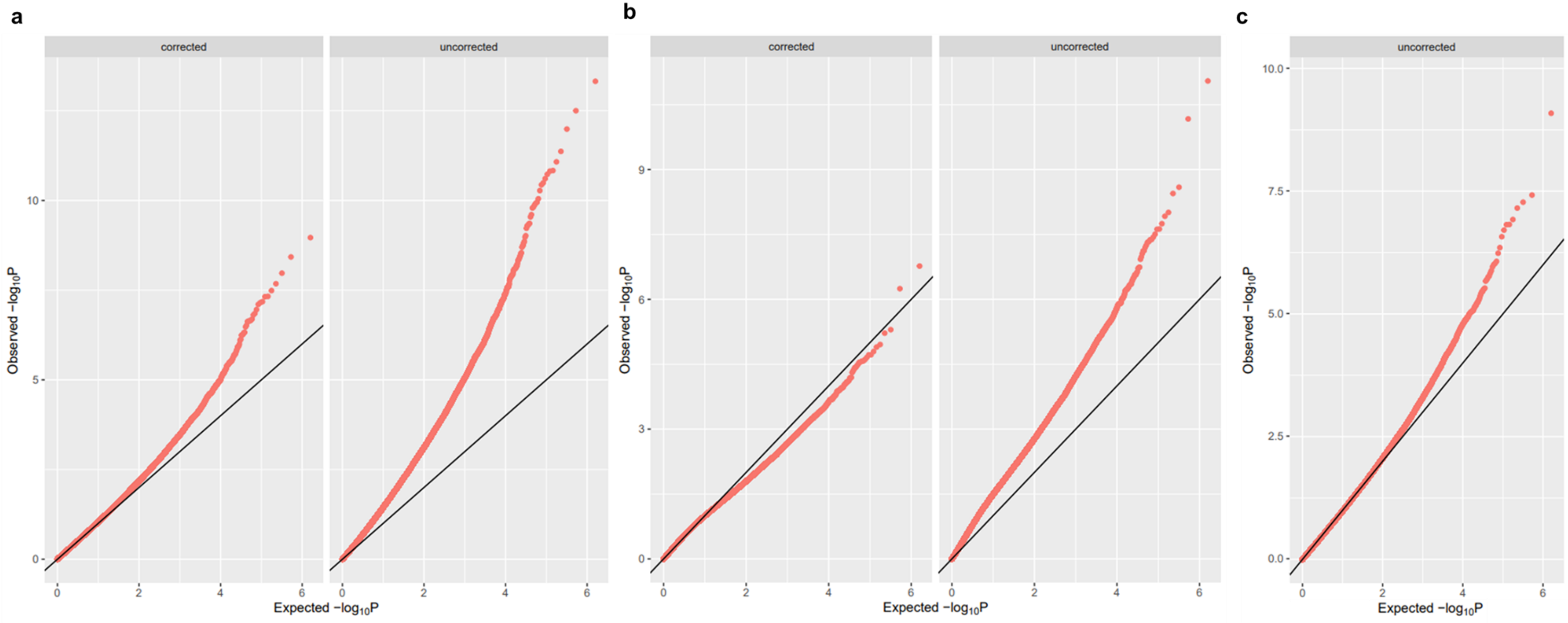

Supplementary Figure 2: Quantile-Quantile (QQ) analysis of *P*-values. QQ plots of the correlation between the expected and observed –log10(*P*) in (**a**), the striatum, and (**b**), the entorhinal cortex, showing Bacon correction (left panels: (**a**) lambda = 1.04, (**b**) lambda = 1.17) and before Bacon correction (right panels: (**a**) lambda = 1.55, (**b**) lambda = 1.92), showing the removal of inflation after correction. (**c**) QQ plot of the correlation between the expected and observed –log10 *P* in the cerebellum, shows no inflation and therefore was not subjected to bacon correction (lambda = 0.917). The black lines denote a theoretical, perfect linear relationship (y=x). The red dots represent the observed *P*-values for each site on the methylation array.

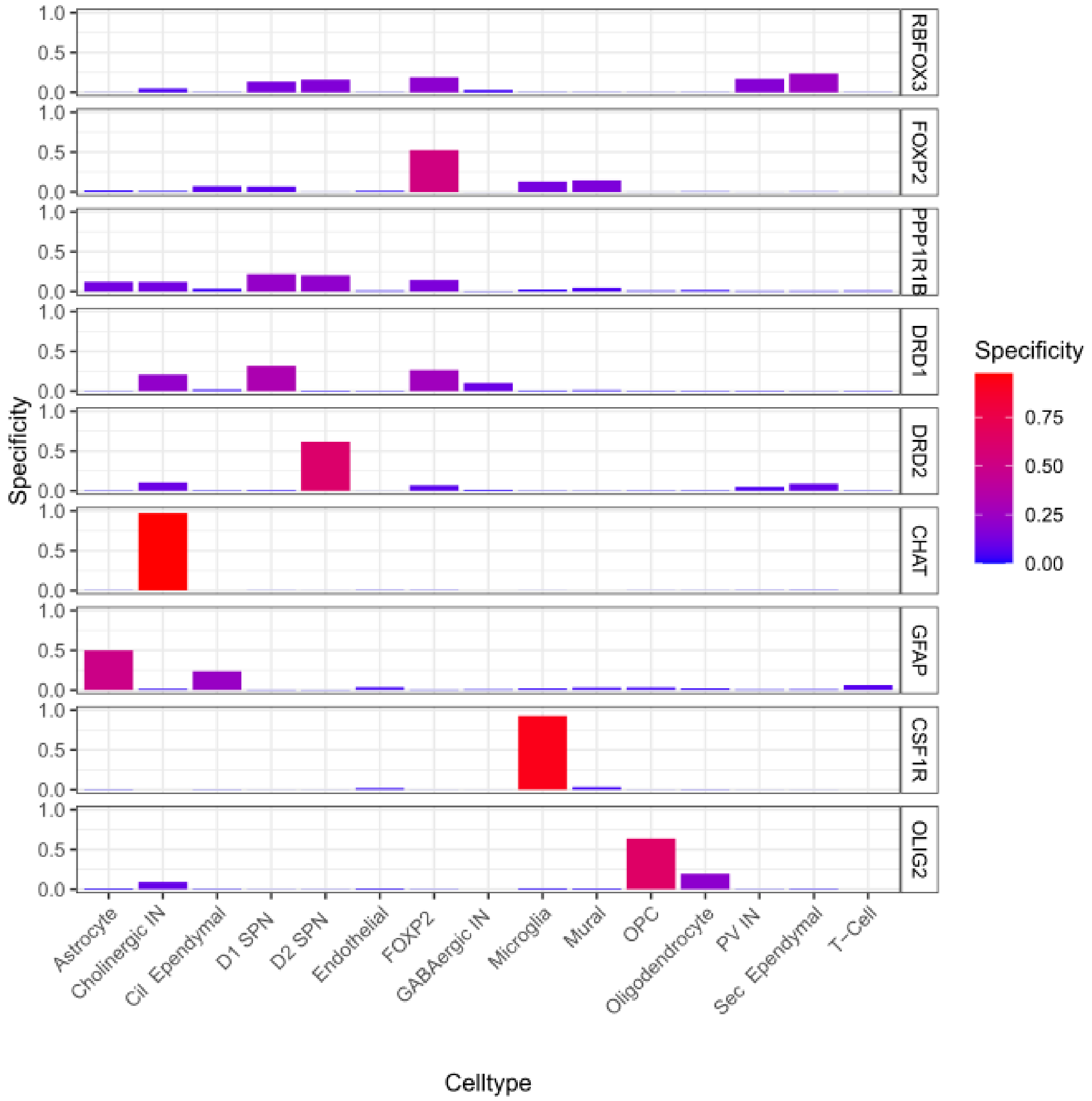

Supplementary Figure 3: Marker gene expression specificity is as expected in the human snRNA-seq dataset used to test cellular enrichment of the HD-associated modules. Specificity of marker gene expression within the different cell types identified by Lee et al., 2020, in the human striatum. Each panel represents the expression of a different marker gene across the cell types. Specificity, denoted as a proportion of the total expression across the cell types is on the y-axis. The x-axis displays the cell types. IN = interneuron, PV = Parvalbumin, Cil Ependymal = cilia ependymal cells and Sec Ependymal = secretory ependymal cells. *RBFOX3* is a neuronal marker. *FOXP2* is a marker of FOXP2 striatal neurons. *PPP1R1B* is a spiny projection neuron marker. *DRD1* is a D1 spiny projection neuron marker. *DRD2* is a D2 spiny projection neuron marker. *CHAT* is a cholinergic IN marker. *GFAP* is predominantly a marker of astrocytes. *CSF1R* is a microglia marker. *OLIG2* is a marker of oligodendrocyte-lineage cells.

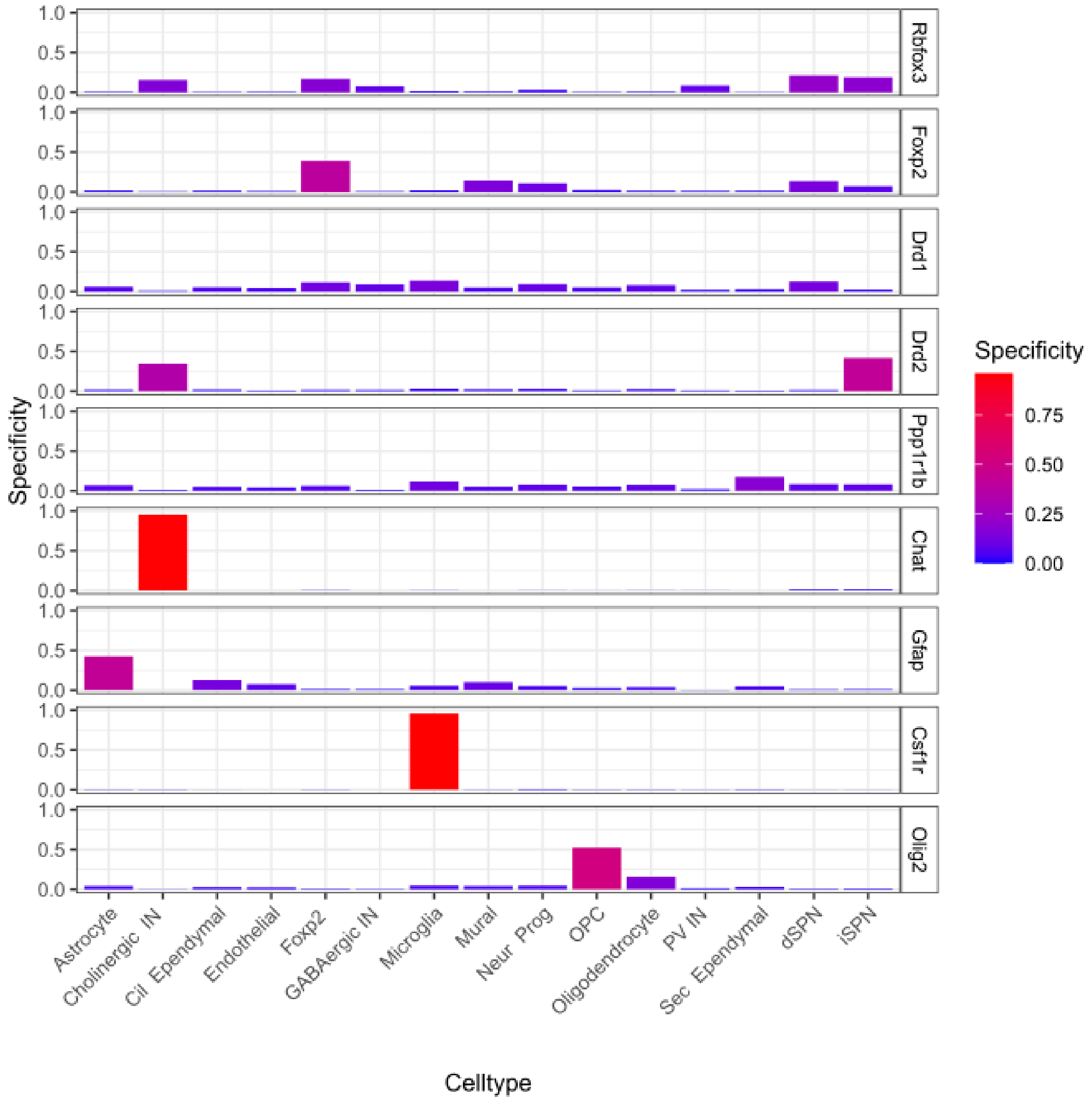

Supplementary Figure 4: Marker gene expression specificity is as expected in the mouse snRNAseq dataset used to test cellular enrichment of the HD-associated modules. Specificity of marker gene expression within the different cell types identified by Lee et al., 2020, in the mouse striatum. Each panel represents the expression of a different marker gene across the cell types. Specificity, denoted as a proportion of the total expression across the cell types is on the y-axis. The x-axis displays the cell types. IN = interneuron, PV = Parvalbumin, ‘Cil Ependymal’ = cilia ependymal cells, ‘Sec Ependymal’ = secretory ependymal cells and ‘Neur Prog’ = neural progenitor cells. *Rbfox3* is a neuronal marker. *Foxp2* is a marker of Foxp2 striatal neurons. *Ppp1r1b* is a spiny projection neuron marker. *Drd1* is a D1 spiny projection neuron marker. *Drd2* is a D2 spiny projection neuron marker. *Chat* is a cholinergic IN marker. *Gfap* is predominantly a marker of astrocytes. *Csf1r* is a microglia marker. *Olig2* is a marker of oligodendrocyte-lineage cells.

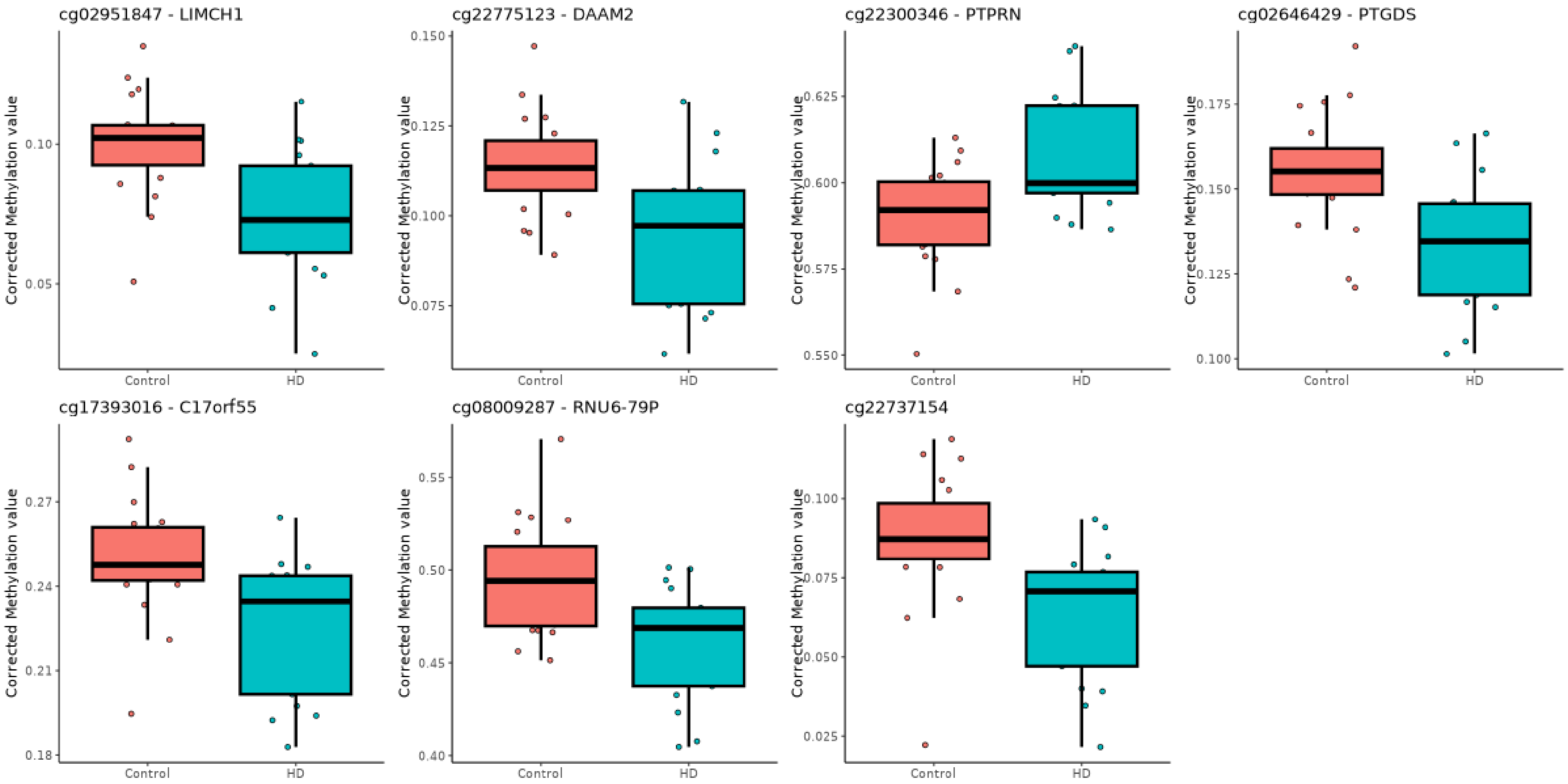

Supplementary Figure 5: CpG sites displaying Bonferroni-significant differential methylation in the striatum in HD. Boxplots of the corrected methylation values (y-axis) for the seven Bonferroni-significant loci in control samples (red) compared to HD samples (blue). The thick black line represents the median value. The boxes represent the middle 50% of values and the whiskers represent the 1st and 4th quartiles. The individual data points are shown to illustrate the distribution of the data. CpGs are labelled with the Illumina ID and UCSC annotated gene.

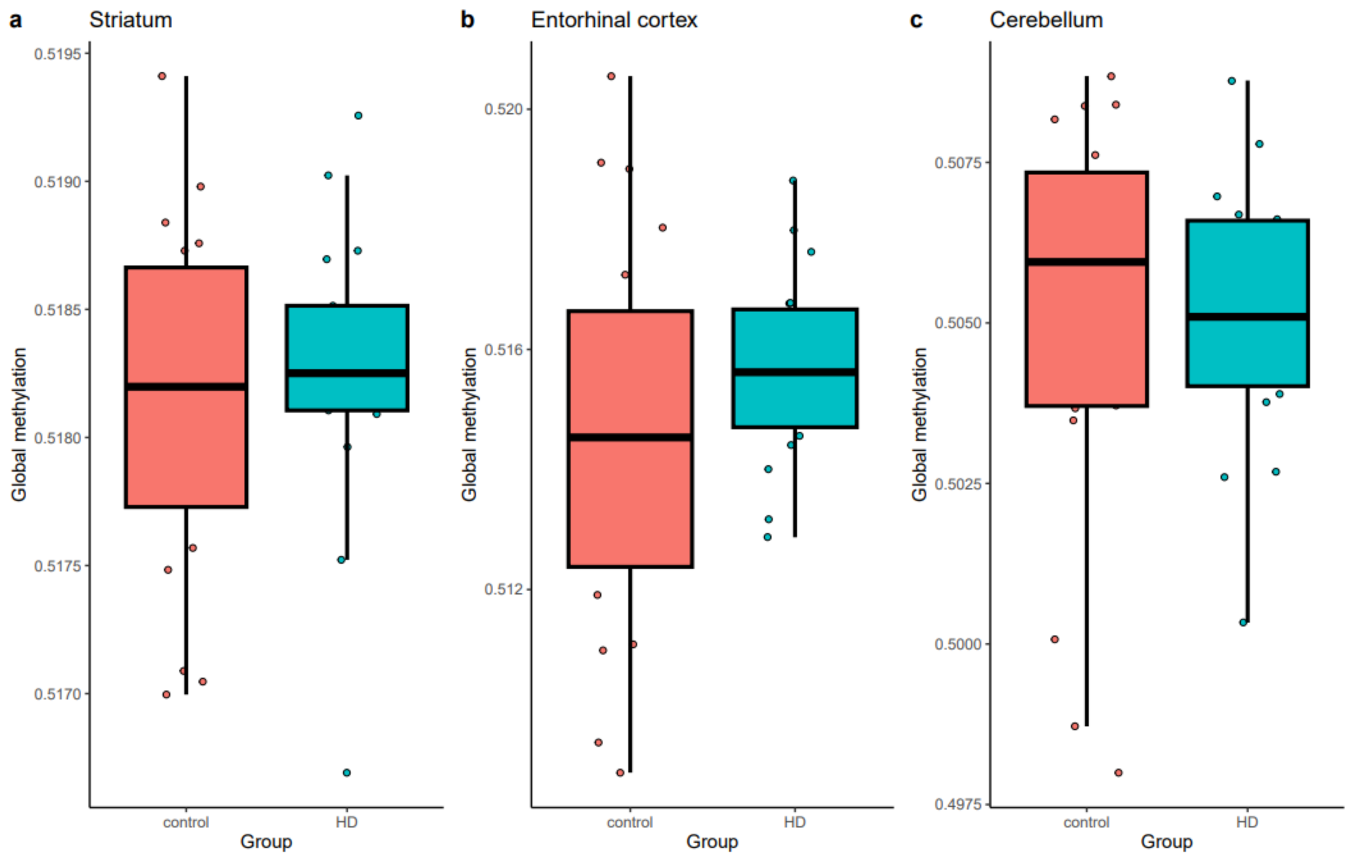

Supplementary Figure 6: No difference in global methylation is observed in HD. Boxplots of the corrected mean global methylation values (y-axis) for control samples (red) compared to HD samples (blue) in (**a**) the striatum, (**b**) the entorhinal cortex and (**c**) the cerebellum. The thick black line represents the median value. The boxes represent the middle 50% of values and the whiskers represent the 1st and 4th quartiles. The individual data points are shown to illustrate the distribution of the data.

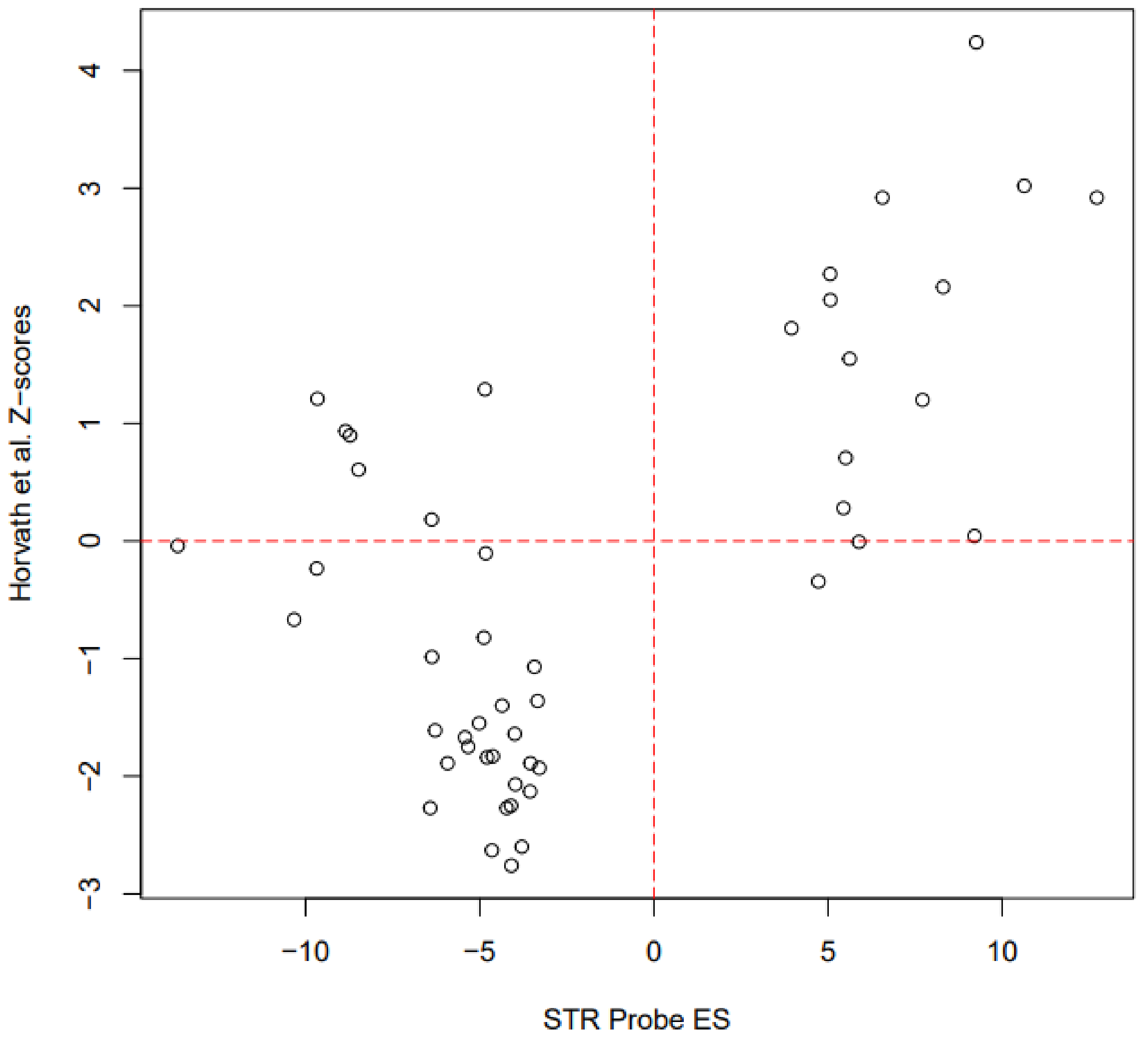

Supplementary Figure 7: The effect size of the most significant sites we identified in the striatum correlated with Z-scores from a previous meta-analysis of frontal, occipital and parietal cortex in HD and control brain samples. Of the 100 most significant DMPs from our EWAS of striatum, 48 of these were present in the summary statistics available from the meta-analysis of multiple brain regions in HD performed by Horvath et al. 2016. Shown is a scatter plot of the effect size (ES) of these 48 CpGs in our striatum (STR) samples (x-axis) against the Z-scores in the meta-analysis of the frontal, occipital and parietal cortices (y-axis). Probes in the bottom left and upper right quadrants, as denoted by the dotted redlines, indicate CpGs with the same direction of effect between the two studies.

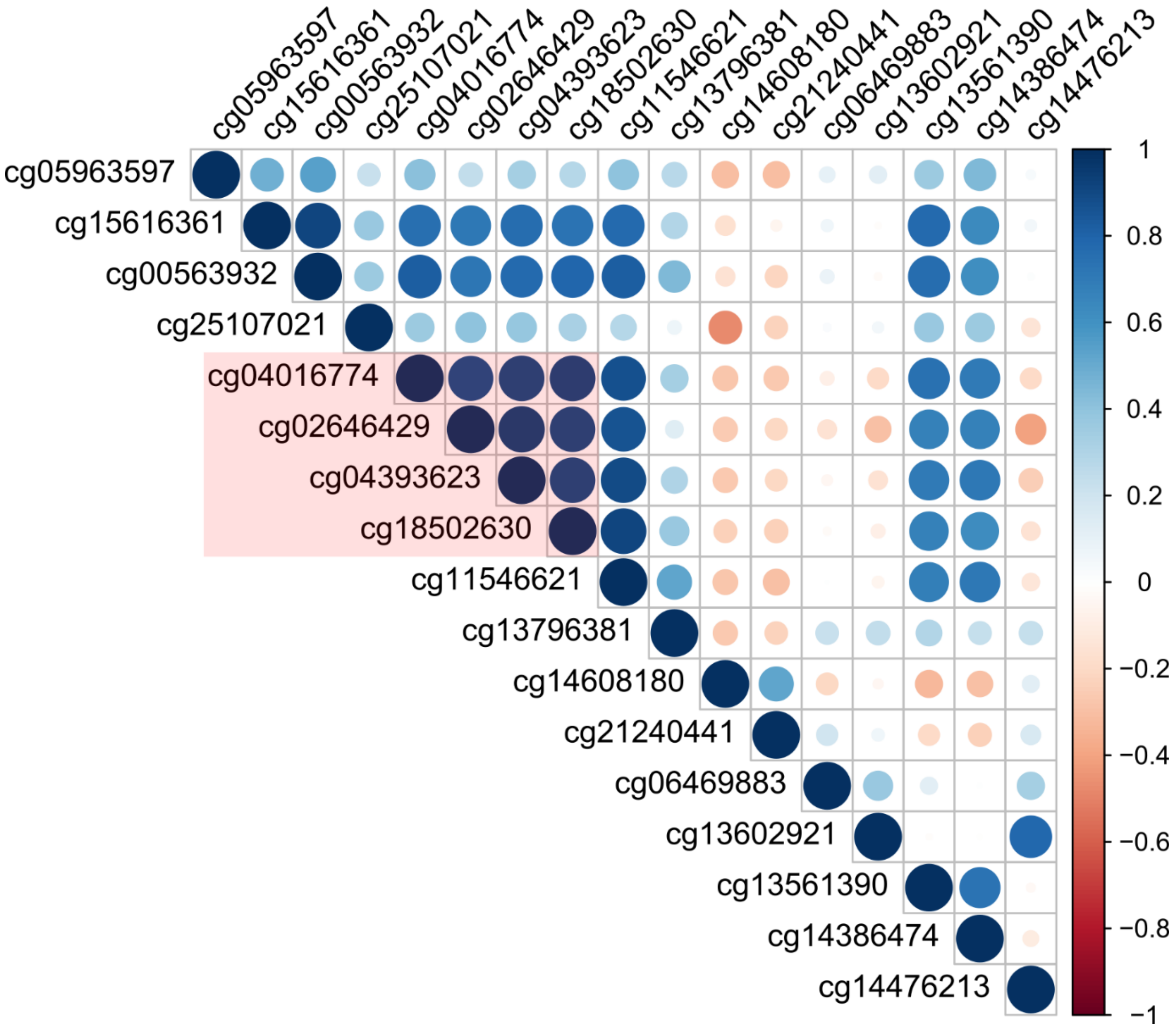

Supplementary Figure 8: DNA methylation levels of probes within the *PTGDS* gene are highly correlated. Correlation plot of the methylation values of CpG sites in the *PTGDS* genomic region that are present on the EPIC array. The 17 sites on the EPIC array contained within the Gencode transcript ENST00000954960.1_1 (*PTGDS*), spanning 6.66 kb, were correlated using Spearman's correlation. The CpGs are ordered by genomic position. Deepening blue circles represent stronger positive correlations, whilst deepening red correlations represent stronger negative correlations. The size of the circles are also proportional to the strength of the correlation. The shaded red box highlights the correlations between the sites within the DMR we identified in the *PTGDS* gene.

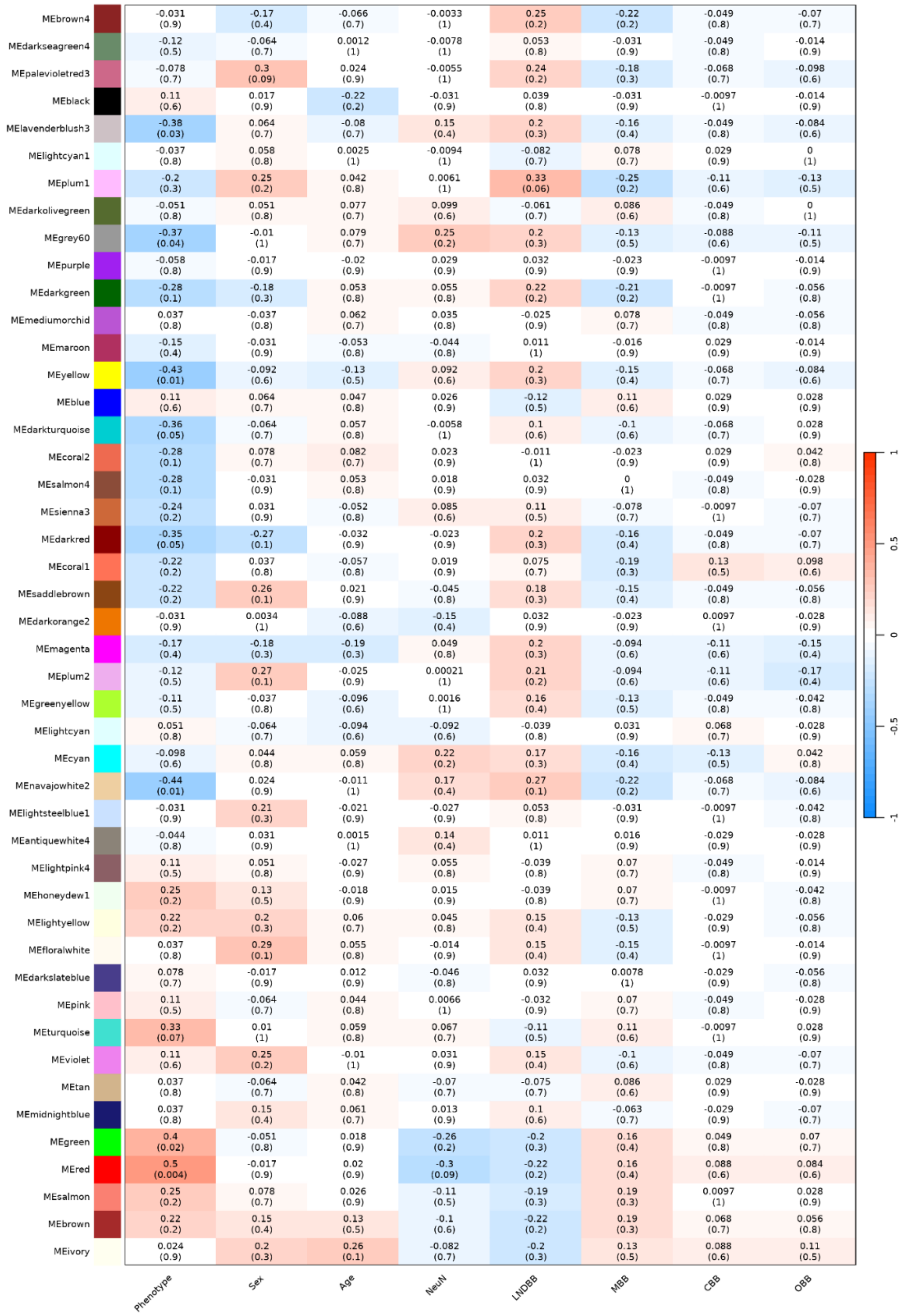

Supplementary Figure 9: Module-trait correlations reveal six modules that are significantly correlated with HD and not with confounders in the striatum. Heatmap of module-trait relationships showing results of Pearson correlation (binary traits) and Spearman correlation (continuous traits). The module name and colour are displayed on the y-axis, whilst the trait of interest is on the x-axis. Values in the boxes represent the correlation score (*r*) with the significance (*P*) in brackets underneath. Darker red boxes represent a stronger positive correlation, whilst deepening blue boxes represent a stronger negative correlation.

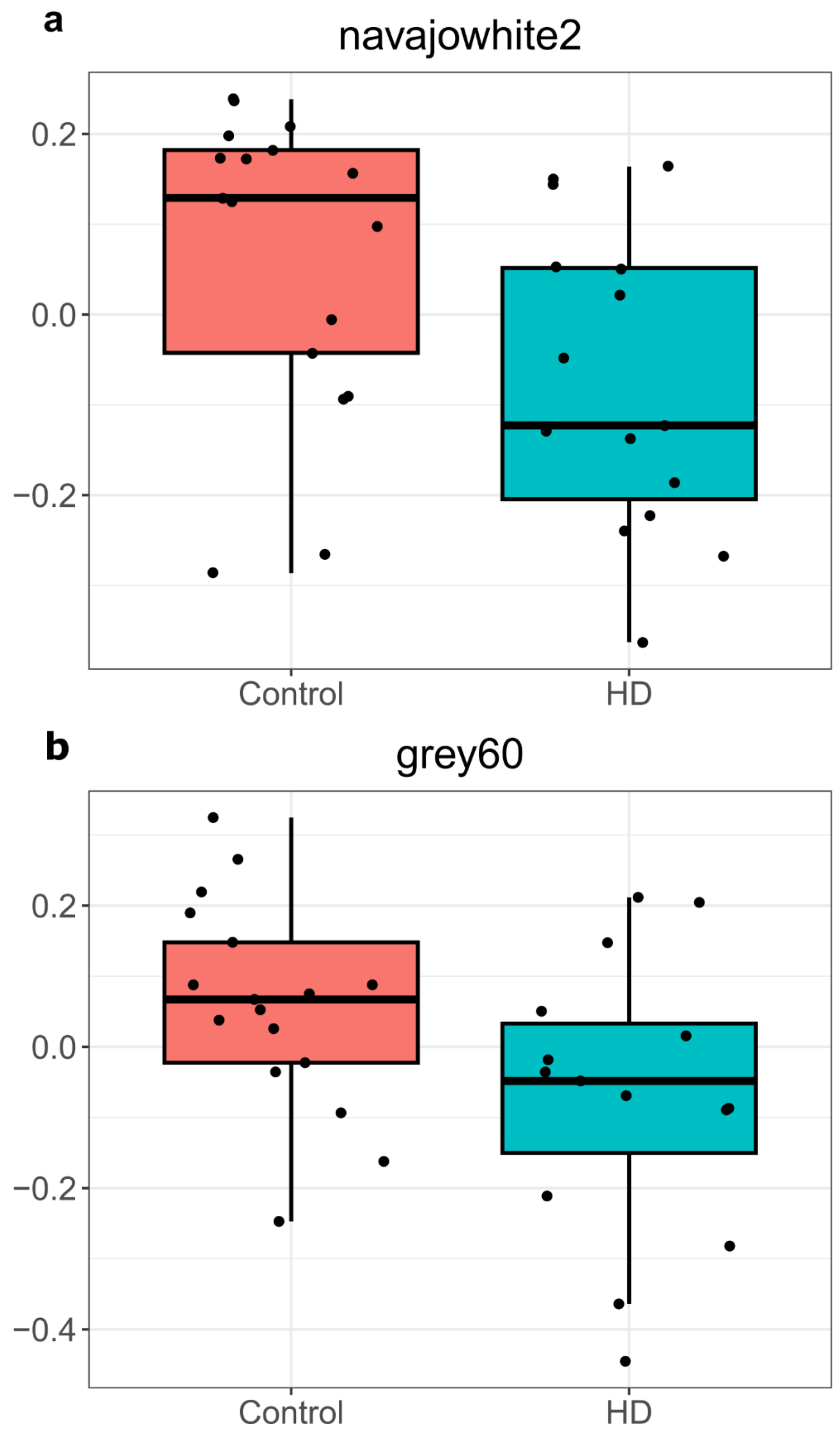

Supplementary Figure 10: Significant differences were observed in the ME values between control and HD samples in HD-associated modules not taken forward for further analysis. Boxplots of the ME value (y-axis) for the control (red) and HD (blue) groups in (**a**) the navajowhite2 (P = 0.0229) and (**b**) grey60 modules (P = 0.0468). The thick black line represents the median value. The boxes represent the middle 50% of values and the whiskers represent the 1st and 4th quartiles.

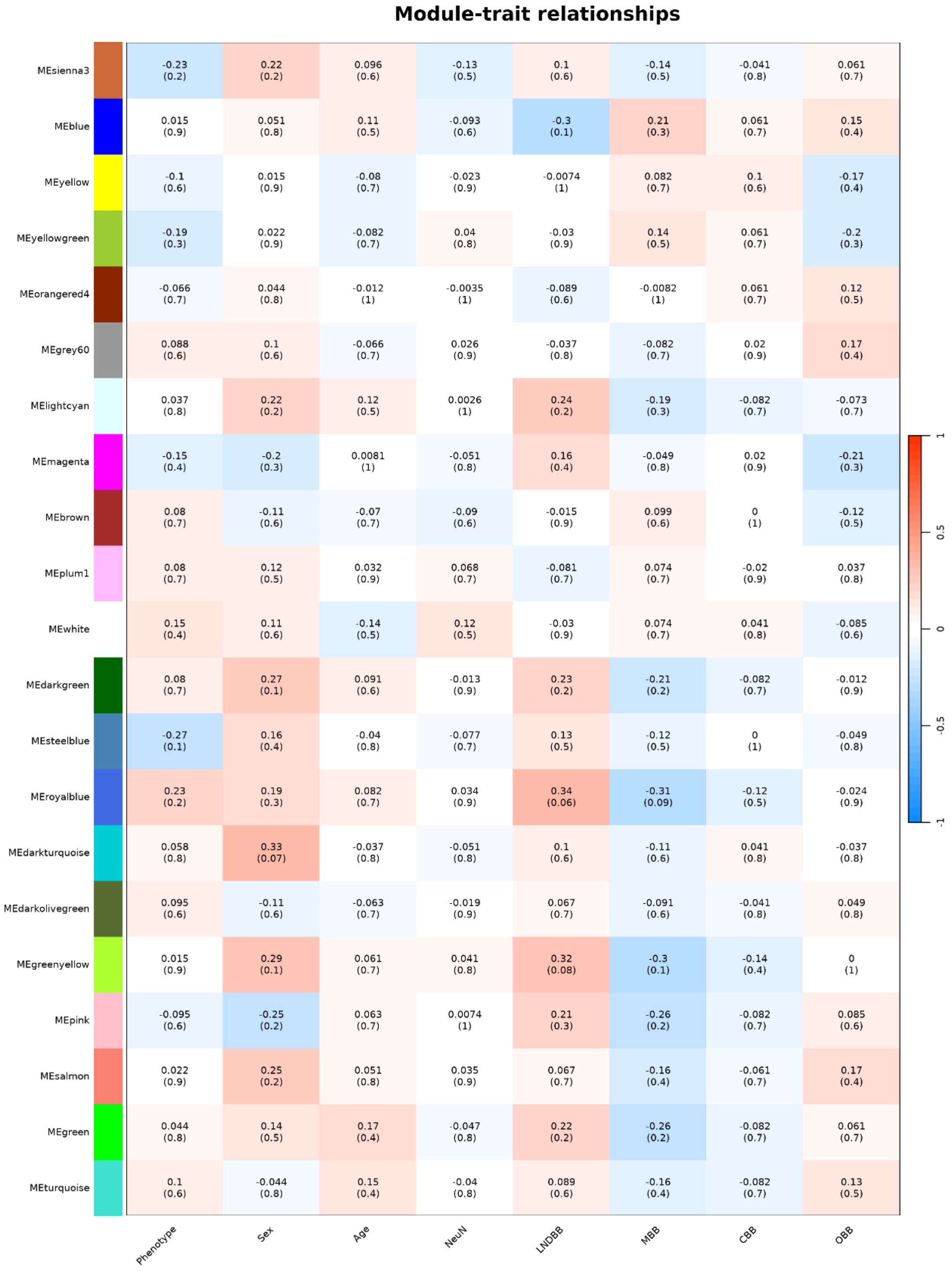

Supplementary Figure 11: Module-trait correlations reveal no modules are significantly correlated with HD status in the entorhinal cortex. Heatmap of module-trait relationships showing results of Pearson correlation (binary traits) and Spearman correlation (continuous traits). The module name and colour are displayed on the y-axis, whilst the trait of interest is on the x-axis. Values in the boxes represent the correlation score (*r*) with the significance (*P*) in brackets underneath. Darker red boxes represent a stronger positive correlation, whilst deepening blue boxes represent a stronger negative correlation.

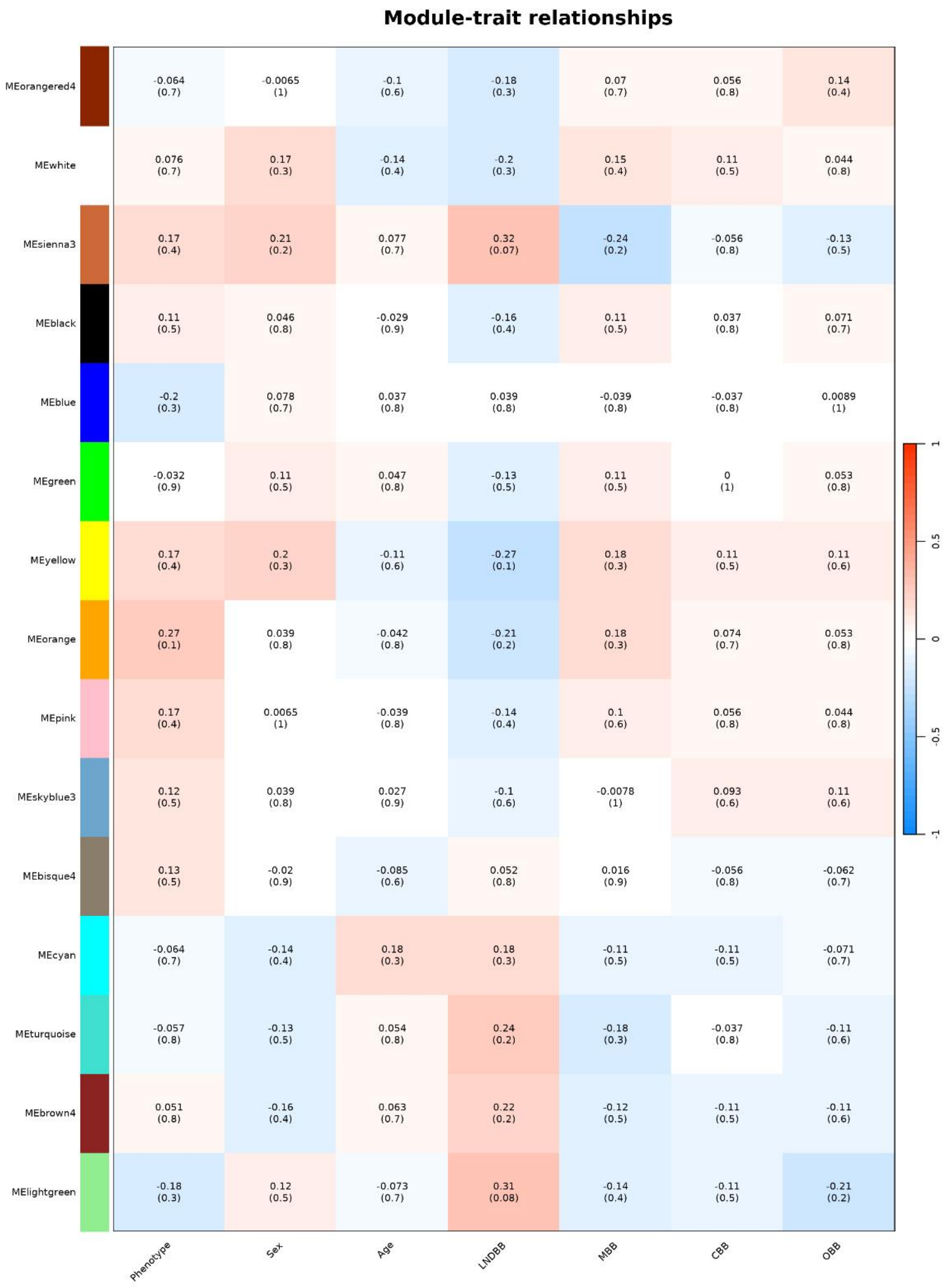

Supplementary Figure 12: Module-trait correlations reveal no modules are significantly correlated with HD status in the cerebellum. Heatmap of module-trait relationships showing results of Pearson correlation (binary traits) and Spearman correlation (continuous traits). The module name and colour are displayed on the y-axis, whilst the trait of interest is on the x-axis. Values in the boxes represent the correlation score (*r*) with the significance (*P*) in brackets underneath. Darker red boxes represent a stronger positive correlation, whilst deepening blue boxes represent a stronger negative correlation.

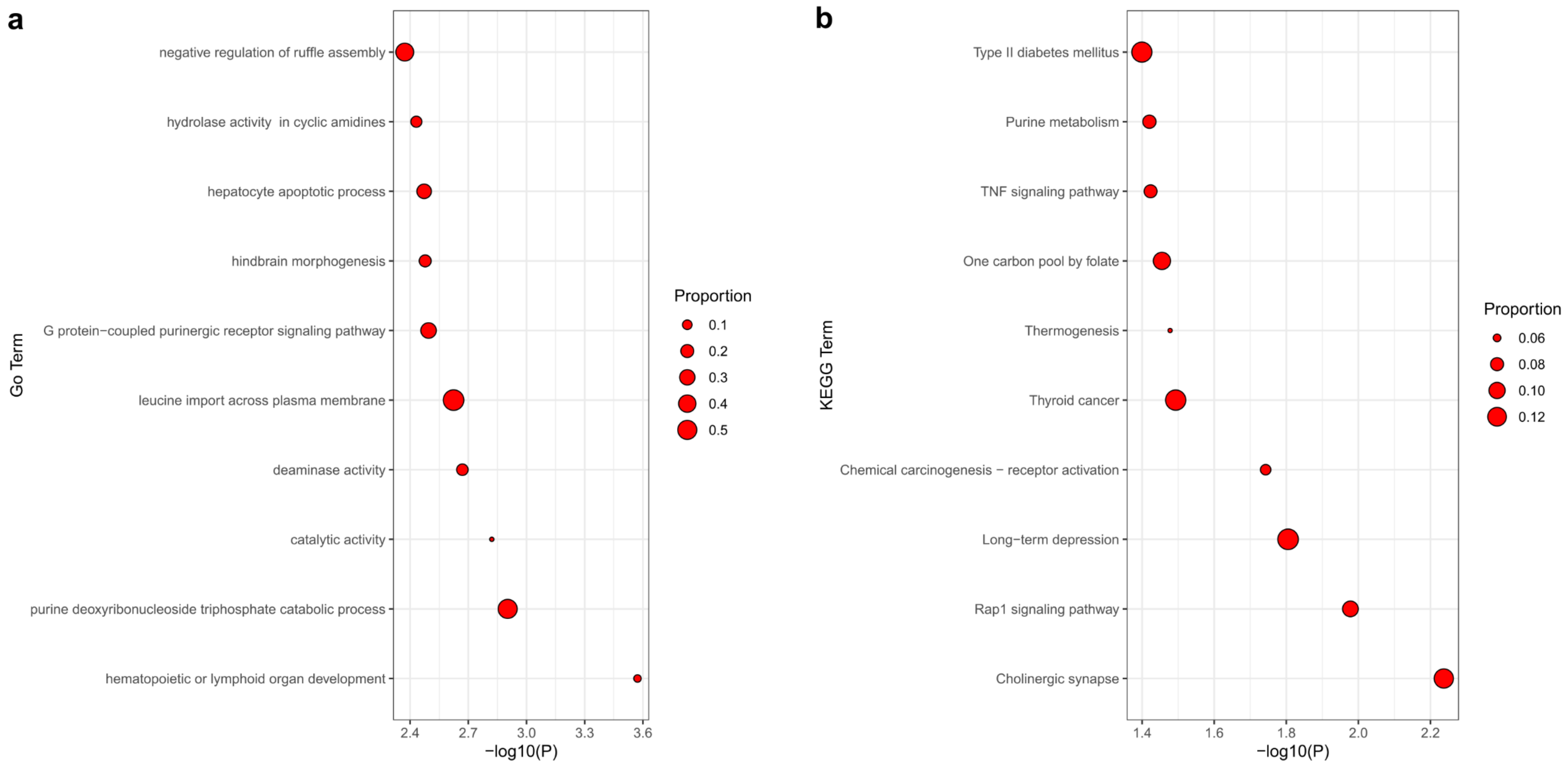

Supplementary Figure 13: GO and KEGG pathway analysis on red module hub probes. Shown are the top 10 most significant terms for (**a**) GO enrichment and (**b**) KEGG pathway analyses for the hub probes (N = 1,064) in the red module, which was significantly associated with HD. The terms are arranged from least significant to most significant. The x-axis displays the -log10(*P*). Points are sized by the proportion of CpGs in the total sites annotated to that term that are part of the red module.

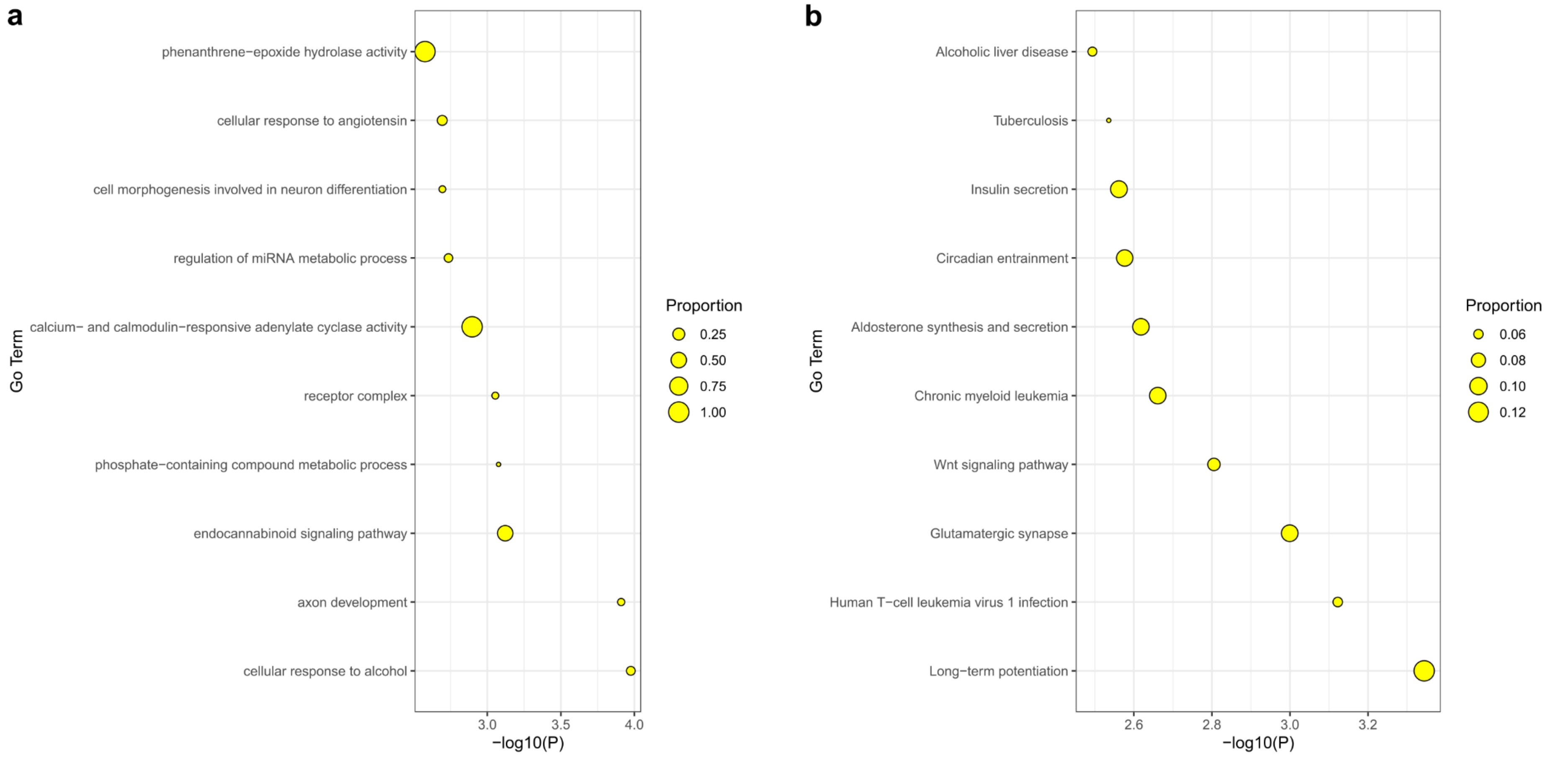

Supplementary Figure 14: GO and KEGG pathway analysis on yellow module hub genes. Shown are the top 10 most significant terms for (**a**) GO enrichment and (**b**) KEGG pathway analyses for the hub probes (N = 454) of the yellow module, which was significantly associated with HD. The terms are arranged from least significant to most significant. The x-axis displays the -log10(*P*). Points are sized by the proportion of CpGs in the total sites annotated to that term that are part of the yellow module.

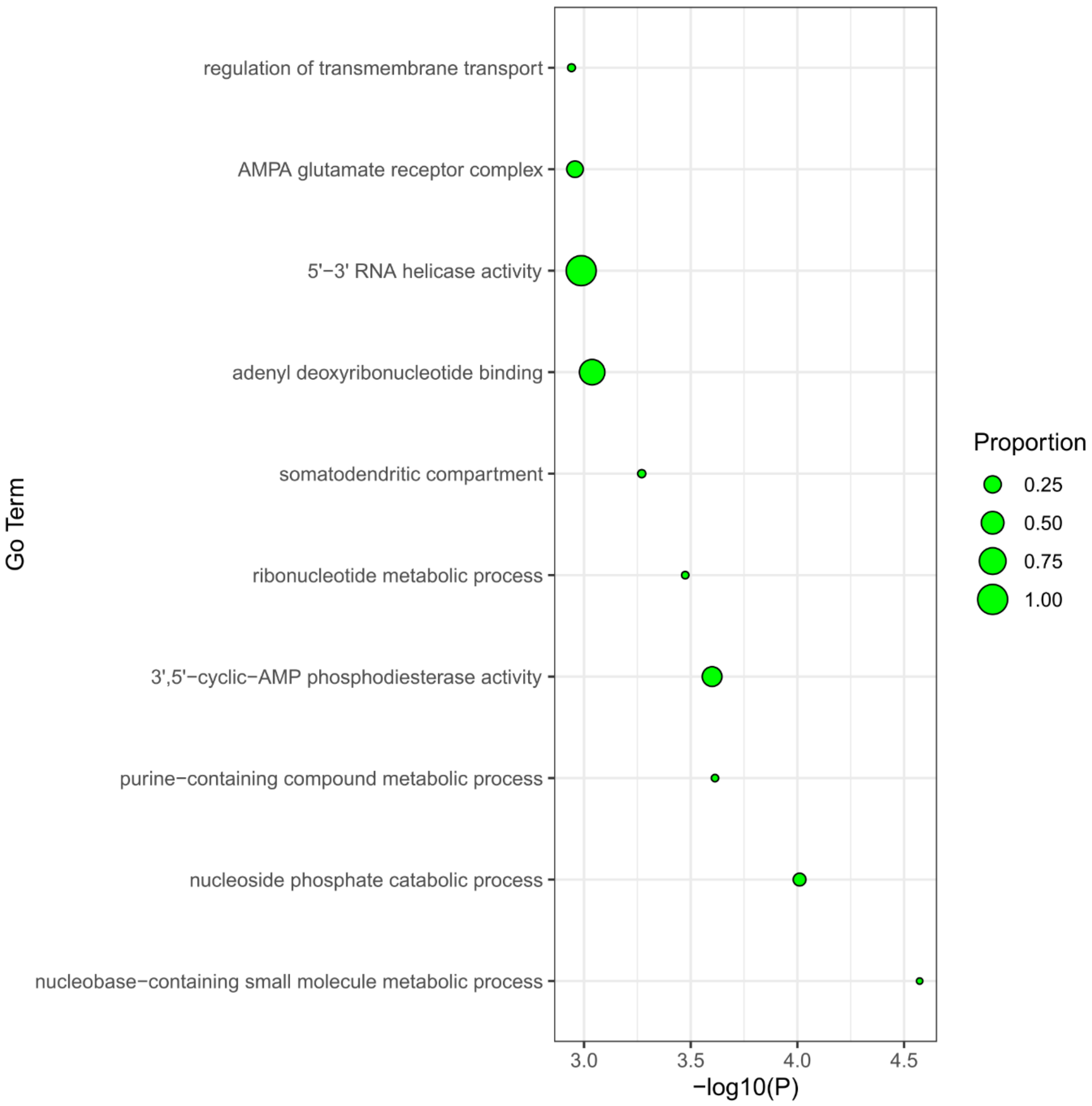

Supplementary Figure 15: GO pathway analysis on green module hub probes. Shown are the top 10 most significant terms for GO enrichment analysis for the hub probes (N = 731) in the green module, which was significantly associated with HD. The terms are arranged from least significant to most significant. The x-axis displays the -log10(*P*). Points are sized by the proportion of CpGs in the total sites annotated to that term that are part of the green module.

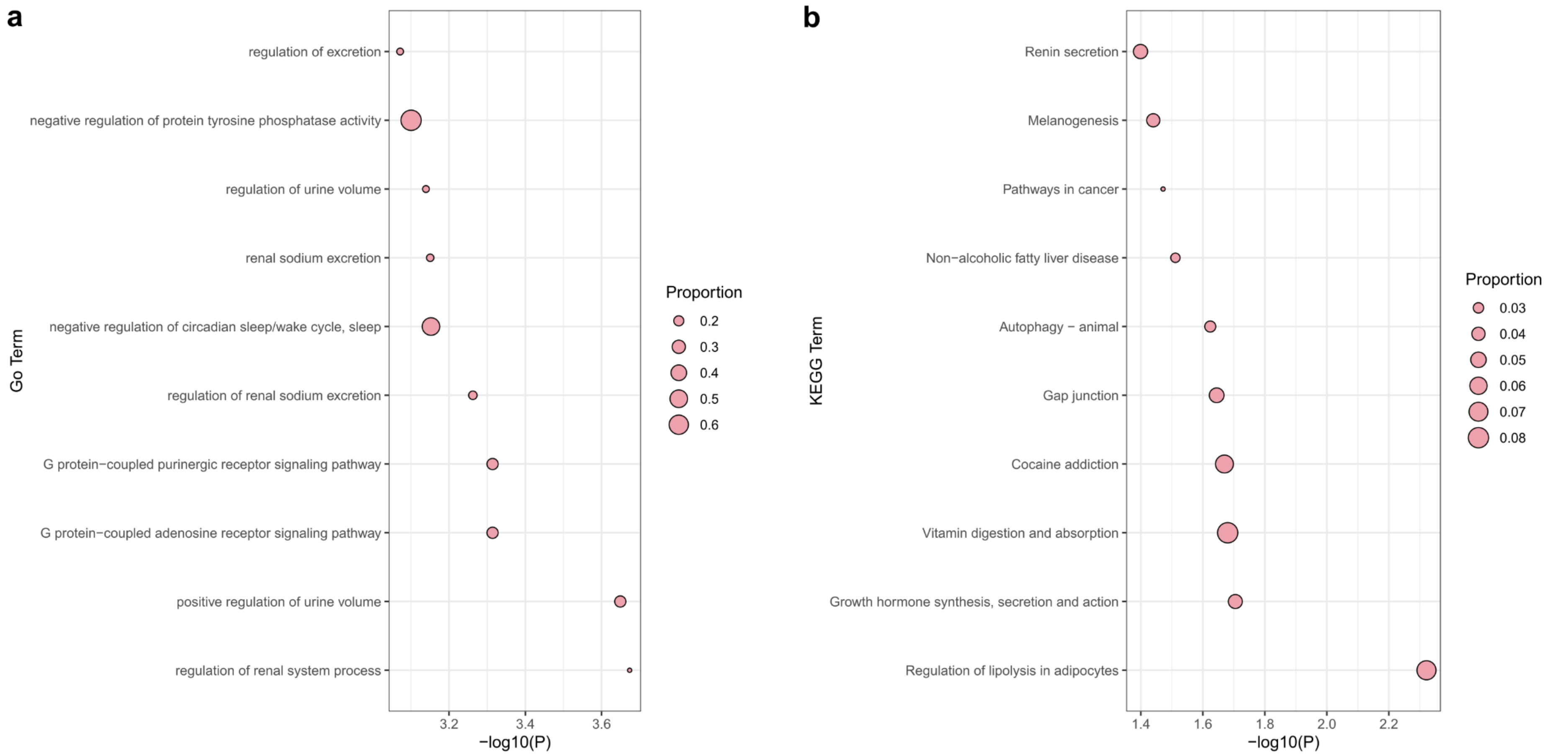

Supplementary Figure 16: GO and KEGG pathway analysis on lavenderblush3 module probes. Shown are the top 10 most significant terms for (**a**) GO enrichment and (**b**) KEGG pathway analyses for the probes in the lavenderblush3 module (N = 454), which was significantly associated with HD. The terms are arranged from least significant to most significant. The x-axis displays the -log10(*P*). Points are sized by the proportion of CpGs in the total sites annotated to that term that are part of the lavenderblush3 module.

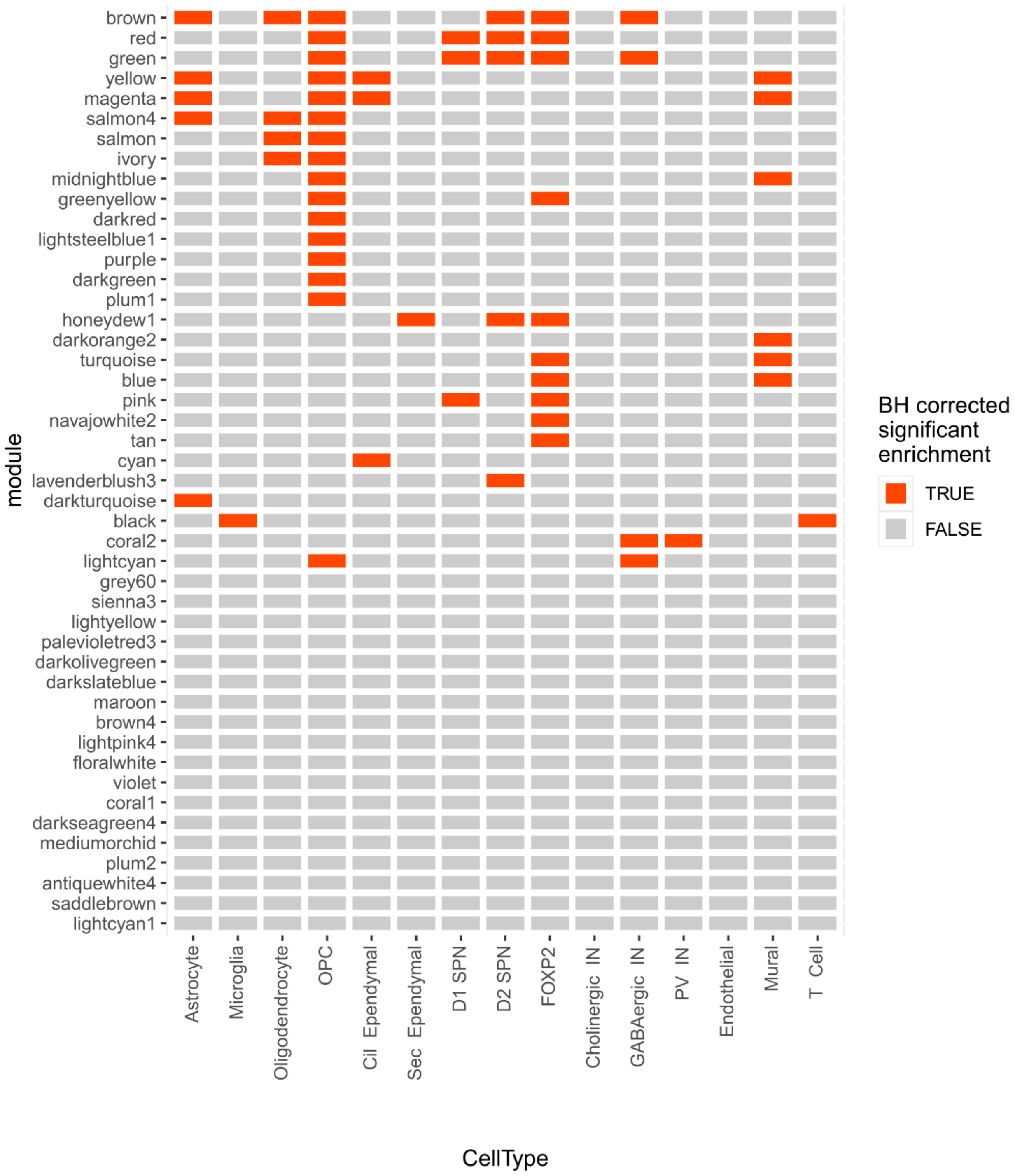

Supplementary Figure 17: Expression weighted cell type enrichment for all 46 filtered modules identified in the striatum. Cells are colored according to the BH corrected *P*-value ($Q < 0.05$) for cell type enrichment, accounting for 690 multiple comparisons across all modules and cell types. The x-axis displays the cell types: IN = interneuron, PV = Parvalbumin, ‘Cil Ependymal’ = cilia ependymal cells and ‘Sec Ependymal’ = secretory ependymal cells. The y-axis displays the module names.

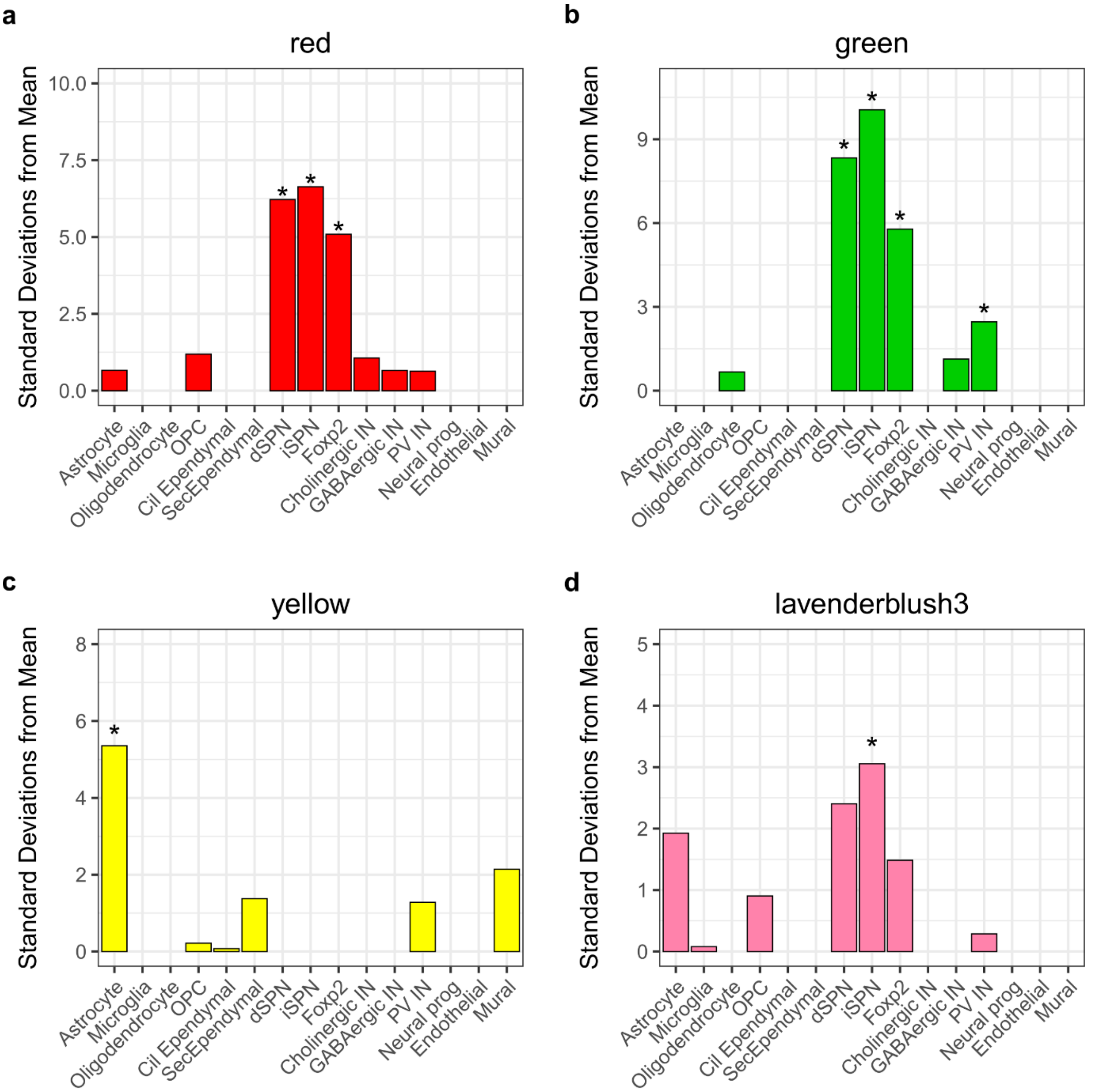

Supplementary Figure 18: Cell type enrichment of genes annotated to the red, green and yellow module hub probes and lavenderblush3 module probes is preserved in the R6/2 HD mouse model. The cell type enrichment of genes annotated to the hub probes in (**a**) the red module, (**b**) the green module, (**c**) the yellow module, and (**d**) all probes in the lavenderblush3 module using a snRNA-seq dataset generated in the striatum of the STR R6/2 HD mouse, obtained from Lee et al., 2020. The x-axis displays the cell type: IN = interneuron, PV = Parvalbumin, 'Cil Ependymal' = cilia ependymal cells, 'Sec Ependymal' = secretory ependymal cells and 'Neural Prog' = neural progenitor cells. BH significant enrichment ($Q < 0.05$) is denoted with an asterisk. The y-axis displays the number of standard deviations from the mean expression for the genes in each module, relative to the bootstrapped mean for the particular cell type.
